# Two mechanisms of cardiac stem cell-mediated cardiomyogenesis in the adult mammalian heart include formation of colonies and cell-in-cell structures

**DOI:** 10.18632/oncotarget.26148

**Published:** 2018-09-25

**Authors:** Galina B. Belostotskaya, Irina V. Nerubatskaya, Michael M. Galagudza

**Affiliations:** ^1^ Sechenov Institute of Evolutionary Physiology and Biochemistry of Russian Academy of Sciences, Russian Federation, Saint-Petersburg, Russian Federation; ^2^ Almazov National Medical Research Centre, Russian Federation, Saint-Petersburg, Russian Federation; ^3^ ITMO University, Russian Federation, Saint-Petersburg, Russian Federation

**Keywords:** cardiac stem cells (CSCs), CSC-derived colony, cell-in-cell structures (CICSs), transitory amplifying cells (TACs), cardiomyogenesis

## Abstract

**Aims:**

Because the mechanism of mature cardiomyocyte (CM) development from cardiac stem cells (CSCs) is not fully understood, we explored the involvement of CSCs into two pathways of cardiomyogenesis in adult mammalian heart: (1) via colony formation and (2) by means of intracellular development of CSCs inside CMs followed by the formation of “cell-in-cell structures” (CICSs).

**Methods and Results:**

Using immunostaining and confocal microscopy, we studied the presence of CSC-derived colonies, CICSs and transitory amplifying cells (TACs), released from ruptured CICSs, in a suspension of *ex vivo* freshly isolated myocardial cells of mammals of different age and species, human including. All subsets of CSCs (c-kit+, Sca-1+ and Isl-1+) were found in mammals of different age. It was shown that c-kit+ and Sca-1+ CSCs produce both colonies and CICSs. However, Isl-1+ CSCs seem to be involved in cardiac growth during first month of age only both through colony formation and CICS generation. In turn, the studies on myocardial cell suspensions of adult C57/bl6N mice, one-year-old bull and 45-year-old woman not only confirmed the involvement of c-kit+ and Sca-1+ CSCs in both mechanisms of cardiomyogenesis, but also showed that Isl-1+ colonies are present in the myocardium of adult mice and rarely in human.

**Conclusions:**

The presence of CSC-derived colonies, CICSs and TACs in all experimental specimens of myocardium proved our previous hypothesis about two pathways that generate new CMs in adult heart. Moreover, we suggest that TACs play a central role in self-renewal of myocardium throughout the lifetime of mammals.

## INTRODUCTION

Current understanding defines the heart as an organ comprised of heterogeneous population of myocytes, which continue to die and self-renew, thereby maintaining cardiac integrity throughout life. However, the cell source for heart self-renewal, purportedly by the replacement of senescent cardiomyocytes (CMs) with juvenile cells, as well as the underlying mechanisms remain obscure. Dedifferentiation of adult CMs [1, 2], transdifferentiation of endogenous stem cells (SCs) [3], and fusion of SCs with cells of other types [4] are usually considered as major scenarios whereby new CMs are generated in adult heart [5]. The concept of mature pre-existing CM division as well as proliferation and differentiation of resident cardiac stem cells (CSCs) are commonly regarded as potential mechanisms of cardiomyogenesis [5, 6]. Along with above described mechanisms, the existence of small CM-like cells gaining the ability to contract within 3 days after plating has been demonstrated in cell culture experiments [7]. Moreover, hypoxia [8] and ischemia [9] have been shown to stimulate proliferation of rare small cardiac marker-positive cells, which are nonetheless unable to regenerate injured myocardium. These observations raise several important questions, including the origin of these small CM-like cells, their localization in healthy myocardium and their *in vivo* potential to regenerate injured myocardium. This issue merits special attention not only because of its importance for the understanding of basic mechanisms that govern myocardial self-renewal but also because of its clinical implications in cell-based therapy of myocardial infarction and chronic heart failure [10–12].

Our previous data obtained in *in vitro* newborn rat cardiac cell cultures showed conclusively that mature contracting CMs were generated during colony formation from resident CSCs of three subtypes, that is, c-kit^+^, Sca-1^+^ и Isl-1^+^ [13]. In addition, we described a phenomenon of intracellular development of CSCs inside mature CMs with the formation of «cell-in-cell structures» (CICSs) followed by the release of transitory amplifying cells (TACs), positive for CSC antigens and cardiac markers [14]. Since TACs released from the CICS are progressively enlarging, which is paralleled by their cardiomyogenic differentiation, these cells might be considered as precardiomyocytes.

To test the involvement of CSCs in these two pathways of cardiomyogenesis in mammalian heart, we characterized colonies and CICSs from a suspension of *ex vivo* freshly isolated myocardium of mammals of different age and from different species, including humans. The presence of CSC-derived colonies and TACs, released from CSC-derived CICSs in all myocardial specimens, provided additional proof for our previous hypothesis about two principal pathways that generate new CMs in adult heart. Moreover, our data strongly suggest that CSC-derived TACs play a central role in self-renewal of myocardium throughout the lifetime of mammals.

## RESULTS

**Figure 1:**
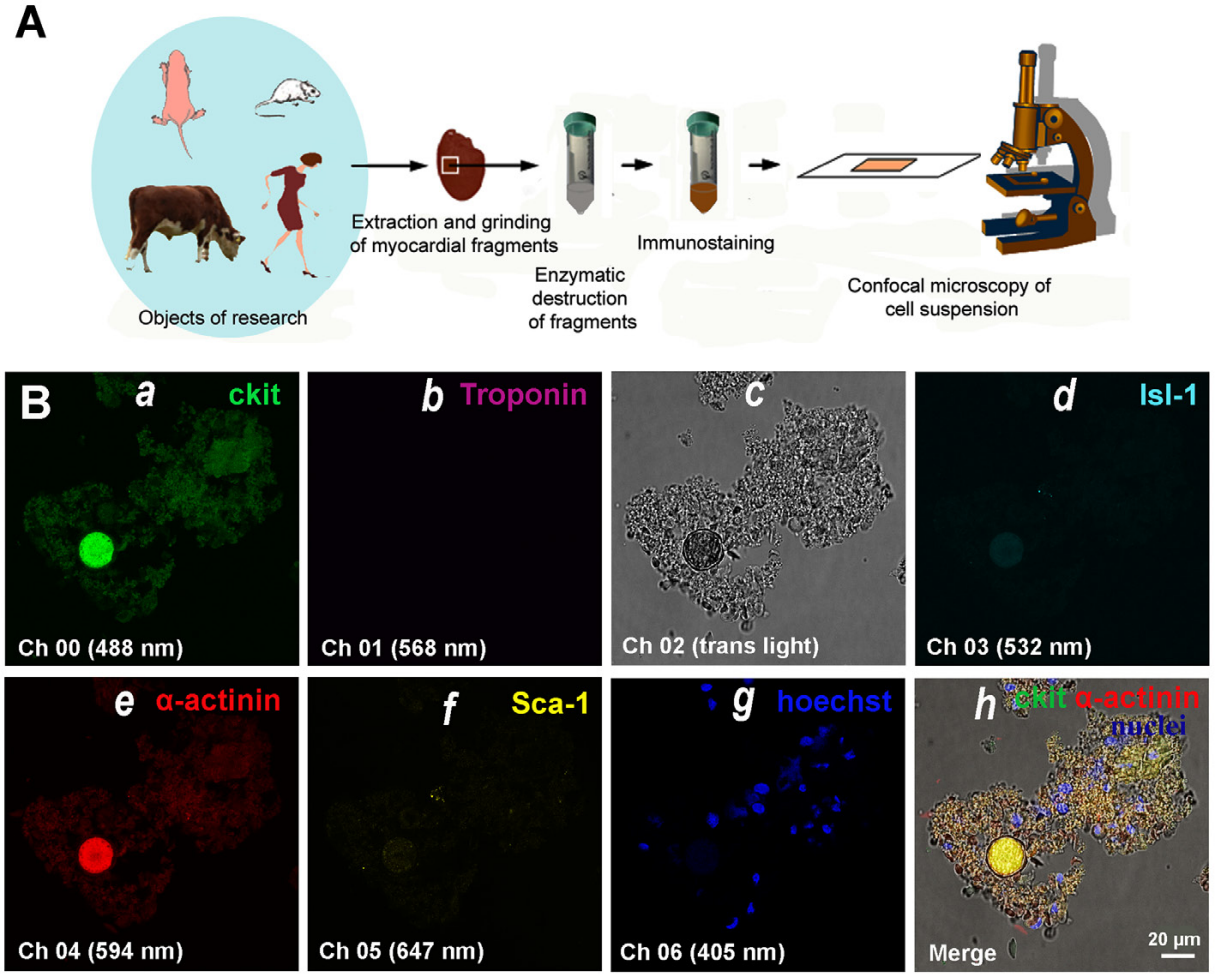
Experimental design **(A)** Dissected hearts or myocardial fragments of mammals of different species and age were enzymatically dissociated into single cell and small fragment suspension. The enzyme-free cell suspension was stained using antibodies, followed by suspending the cells between the slide and cover slip. **(B)** Confocal microscopy of myocardial cells of 4.5-month-old rat. (a) c-kit+. (b) Troponin. (c) Transmitted light. (d) Isl-1+. (e) Sarcomeric α-actinin. (f) Sca-1+. (g) Hoechst (nuclei). (h) Merged c-kit+, Sarcomeric α-actinin, and Hoechst staining on transmitted light image.

Freshly-isolated myocardial cells were fixed, permeabilized, and stained using a set of antibodies against CSCs and cardiac antigens. The design of the experiments are shown in Figure 1. We utilized 6 simultaneous markers: antibodies to cardiac stem cell antigens (c-kit^+^, Isl-1^+^, Sca-1^+^) and cardiac proteins (Sarcomeric a-actinin or a-Sarcomeric actin and Troponin T), and Hoechst for nuclear staining. The highest fluorescence brightness of one of three CSC markers at a single sample, as well as a positive expression of cardiac proteins, was allowed to define what type of CSCs have formed colonies or CICSs (see Materials and Methods, Figure 1B and Supplementary).

**Figure 2:**
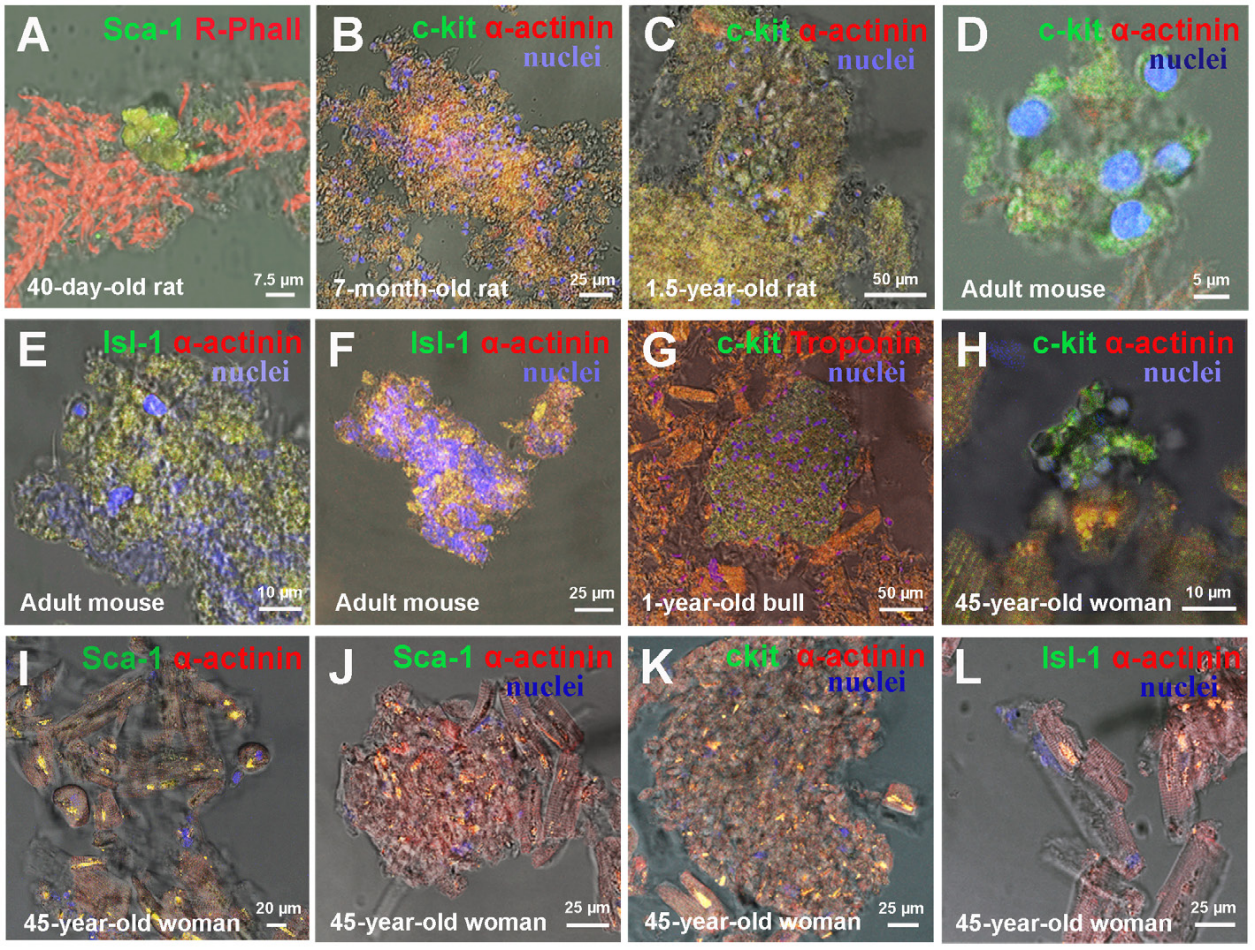
*Ex vivo* characterization of CSC-derived colonies in freshly isolated suspension of myocardial cells of Wistar rats, adultС57bl mouse and 45-years-old woman **(A)** Small colony of 40-day-old rat Sca-1+ CSCs. **(B)** 7-month-old rat c-kit+ colony. **(C)** 1.5-year-old rat c-kit+ colony. **(D)** Adult mouse c-kit+ colony. **(E)** Adult mouse Isl-1+ colony. **(F)** Adult mouse Isl-1+ colony. **(G)** One-year-old bull c-kit+ colony. **(H-L)** 45-year-old woman colonies. c-kit+, Isl-1+, Sca-1+ (green). Rhodamin-Phalloidin,Sarcomeric α-actinin, Troponin T (red). Nuclei (Hoechst), blue.

*Ex vivo* analysis of cell suspension showed that the formation of mature CMs via proliferation and differentiation of CSCs inside the colonies occurs in mammals of different age and different species, including humans. For example, a small colony of immature Sca-1^+^ CSCs (Figure 2A) was identified in a fragment of myocardium derived from a 40-day-old rat, while large c-kit^+^ colonies of various maturity were identified in adult rat myocardium. For example, the colonies were found to be more mature in 7-month-old rats (Figure 2B and Supplementary Figure 1) compared to 1.5-year-old rats (Figure 2C and Supplementary Figure 2). In the hearts of adult C57/bl6N mice we also registered colonies of different level of maturity: an immature c-kit^+^ colony with negligible numbers of CSCs (Figure 2D), a small poorly differentiated Isl-1^+^ colony (Figure 2E) and a large highly differentiated Isl-1^+^ colony, positively stained for Sarcomeric α-actinin (Figure 2F and Supplementary Figure 3). Large poorly differentiated c-kit^+^ colony positive to Troponin T was identified in the myocardium of one-year old steer (Figure 2G and Supplementary Figure 4). Besides, colonies of various sizes and maturity formed by CSCs of three different subtypes were found in the heart of 45-year-old woman (Figure 2H-2L and Supplementary Figure 5, 6). Given the fact that the length of human cardiomyocytes ranges from 50 to 150 μm, we consider the compact clusters of CSC-positive cardiac cells of different sizes and different degrees of differentiation as colonies of developing cardiomyocytes. For example, such colony is shown in Figure 2H and Supplementary Figure 5, consisting from small undifferentiated c-kit^+^ CSCs with diameters ranging from 5.4 to 19.7 μm, while cells inside the compact colony formed by Sca-1^+^ CSCs (Figure 2I and Supplementary Figure 6) are poorly differentiated and have L_mid_ = 46.8 ± 20.6 μm. CMs at the periphery of this colony were found to reach 84.7 μm in length. The images on Figure 2J-2L represent colonies of different maturity, different expression of stem cell antigens and sizes of CMs in adult human heart. The length of cells in compact Sca-1^+^ colony (see Figure 2J) range from 16.3 μm to 67.3 μm (L_mid_= 39 ± 13 μm), while the size of cells inside colony formed by c-kit^+^ CSCs (Figure 2K) varies from 14.6 μm to 68.6 μm (L_mid_ = 29.9 ± 17.7 μm). In turn, mature CMs mildly expressing Isl-1^+^ antigen with L_mid_ = 63.8 ± 18.9 μm and L_max_ reaching 98.4 μm are shown in Figure 2L.

**Figure 3:**
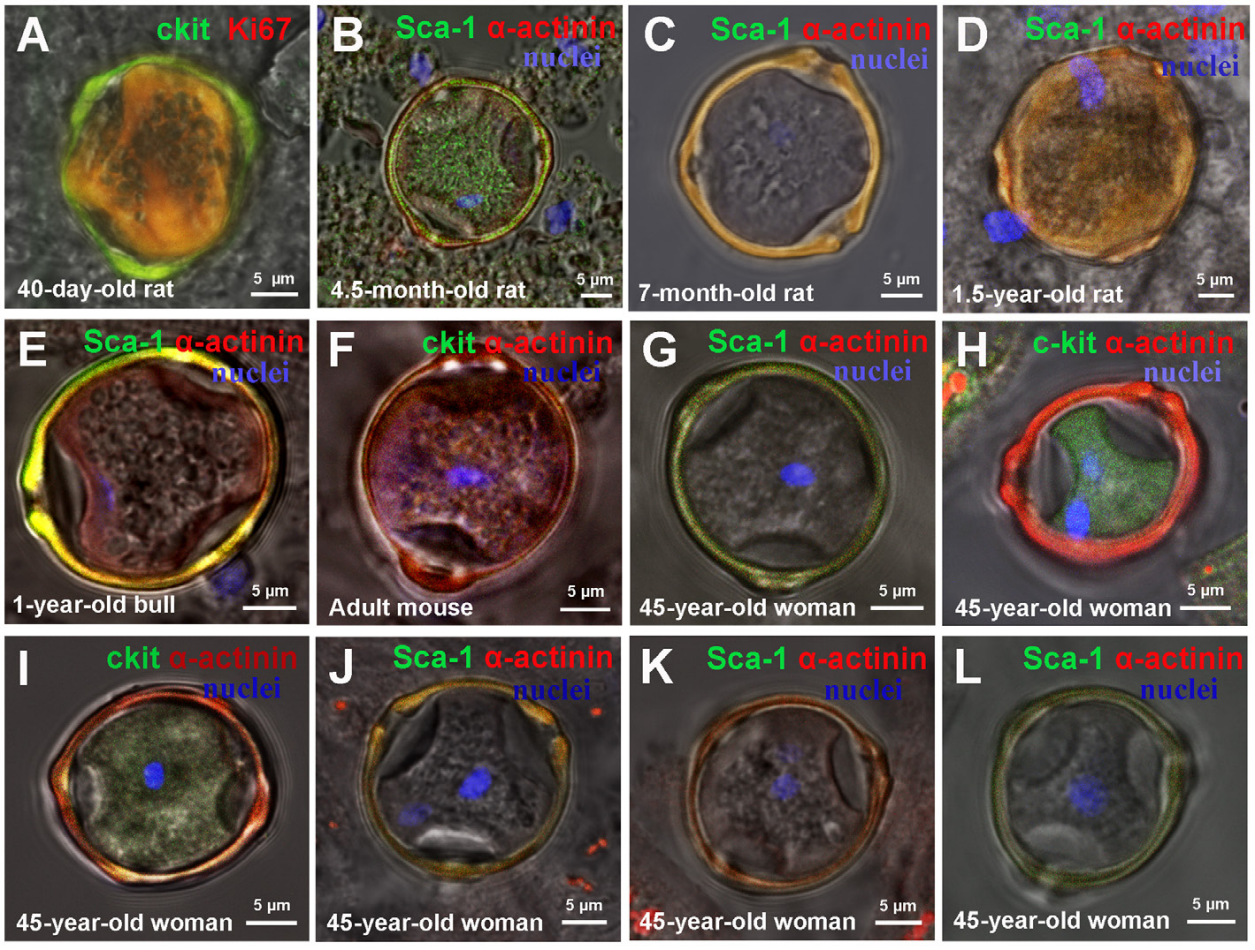
Types of cell-in-cell structures (CICSs) in a suspension of ex vivo freshly-isolated myocardial cells of mammals of different age and species **(A)** 40-day-old rat c-kit+ CICS. **(B)** 4.5-month-old rat Sca-1+CICS. **(C)** 7-month-old rat c-kit+ CICS. **(D)** 1.5-year-old rat Sca-1+ CICS. **(E)** 1-year-old bull Sca-1+ CICS (See also Video 1). **(F)** Adult mouse c-kit+ CICS. **(G-L)** 45-year-old woman CICS (See also Video 2 and Video 3). c-kit+, Isl-1+, Sca-1+ (green). Ki-67-Phycoerythrin, Sarcomeric α-actinin (red). Nuclei (Hoechst), blue.

**Figure 4:**
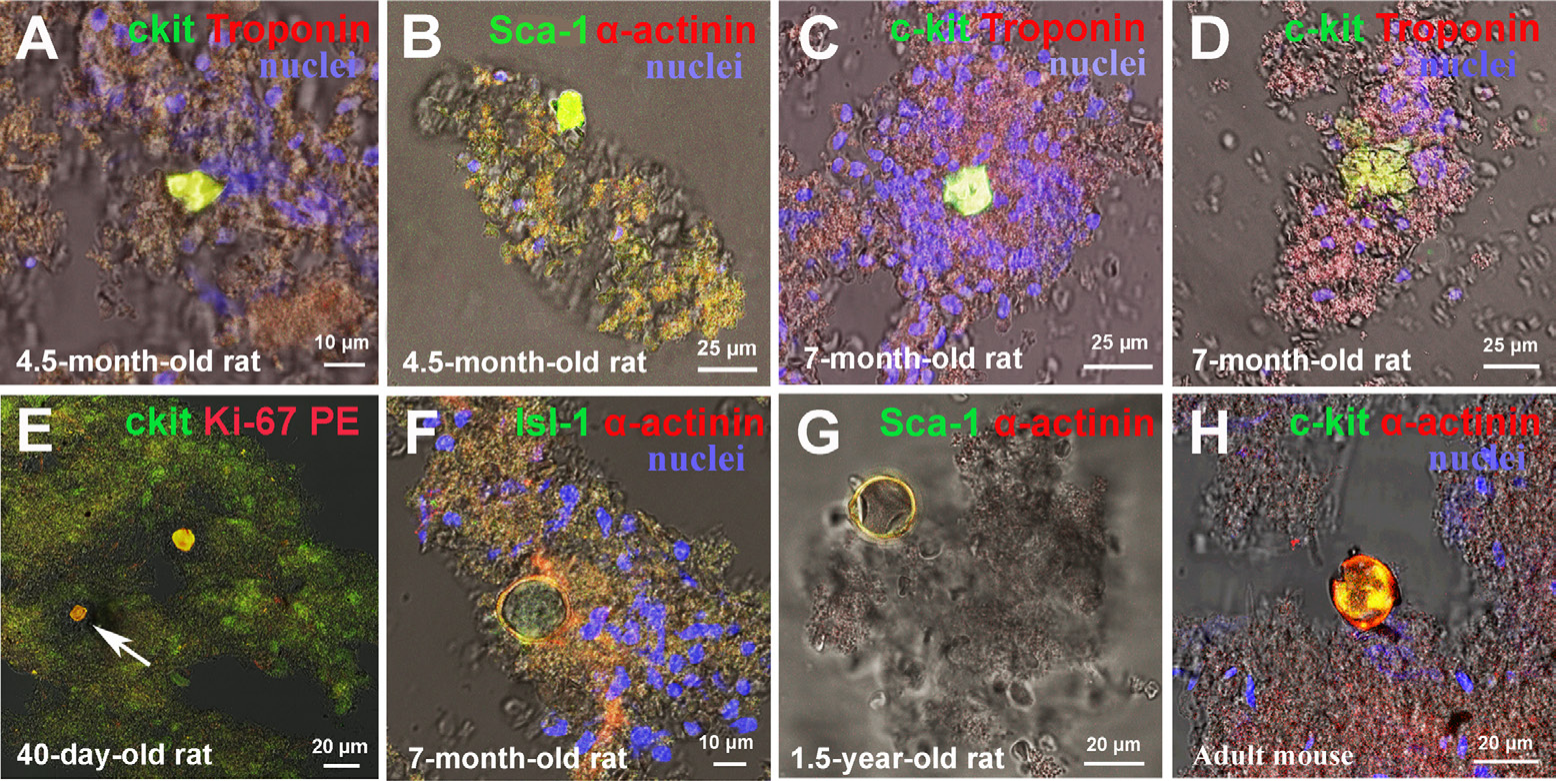
Ex vivo characterization of new colonies and cell-in-cell structures (CICSs) inside previously formed colonies **(A)** 4.5-month-old rat c-kit+ colony. **(B)** 4.5-month-old rat Sca-1+ colony. **(C)** 7-month-old rat c-kit+ colony. **(D)** 7-month-old rat c-kit+ colony. **(E)** 40-day-old rat c-kit+ CICS (see also Video 4). **(F)** 7-month-old rat Isl-1+ CICS. **(G)** 1.5-year-old rat Sca-1+ CICS. **(H)** Adult mouse c-kit+ CICS. c-kit+, Isl-1+, Sca-1+ (green). Ki-67-Phycoerythrin, Sarcomeric α-actinin, Troponin T (red). Nuclei (Hoechst), blue.

Along with identification and characterization of CSC-derived colonies in suspensions of myocardial cells from different mammalian species, we revealed “cell-in-cell structures” (CICSs) formed by c-kit^+^-, Sca-1^+^- and Isl-1^+^ subtypes of CSCs. It was seen that CICSs of different maturity have similar morphology with the presence of dense capsule and well-defined openings (pores) on its surface (Figure 3). The number of pores was found to vary from three in one-year-old steer (Figure 3E, Supplementary Video 1) and 45-year-old woman (Figure 3J, Supplementary Video 2) to four in 45-year-old woman (Figure 3I, Supplementary Video 3) and 40-day-old rat (Figure 4E (arrow), Supplementary Video 4). CICSs with three pores were characterized by spherical form (see Figure 3) while CICSs bearing four pores had more complex tetrahedral form (see Supplementary Video 3 and 4).

CICS sizes (length, width) ranged from 22.5±1.2 μm to 29.7±3.5 μm with vertical dimension 10–41 μm. Notably, D_mid_ were almost identical in young and old mammals of different species, whereas the volumes demonstrated considerable variability from 6211 ± 2185 μm^3^ to 10704 ± 3332 μm^3^, with large standard deviations, which, however, are not statistically significant (Table 1). Moreover, there were no significant interspecies differences in morphology and size of myocardial CICSs compared to CICSs generated in primary culture (Table 2). The data presented in Table 2 show that the volume of CICSs produced over the period of 20 days in primary culture of myocardial cells obtained from newborn rats is similar to that of CICSs from the myocardium of juvenile, adult and old rats (Table 1). Moreover, the volume of CICSs was shown to be almost the same in all mammalian species investigated, taking into account the interspecies differences in cardiomyocyte dimensions.

**Table 1:** CICS parameters of mammals of different species and ages in *ex vivo* experiments

Animals	D_mid_ (μm)	V (μm3)
20-day-old rats	25.8±0.3	8258±1440
40-day-old rats	26.1±2.5	8469±3229
4.5-month-old rats	26.4±9.0	6241±2720
7-month-old rats	25.1±1.9	7480±2480
1,5-year-old rats	23.8±3.2	7061±2997
1-year-old bull	25.4±0.8	8763±511
Adult mice	25.1±2.0	10704±3332
45-year-old woman	22.2±1.5	6211±2185

**Table 2:** The change of CICS sizes during 20 days in the culture of cardiac cells of newborn rats (*in vitro*)

Day *In Vitro*	D_mid_ (μm)	V (μm3)
6-11	26.3±7.2	6972±3552
16-20	26.8±2.8	9061±4598

The middle диаметр (D_mid_) and the cell volume (V) were calculated, using the following formula: V = 3.14 / 6 × L × W × vertical dimension. D_mid_ = (L + W) / 2, where L – length, W – width.

The fact that maximum volume of intact CICSs did not exceed 15000 μm^3^ with mean value of 8000 μm^3^ suggests that intracellular CSC development occurs in incompletely differentiated cardiac myocytes rather than in mature cardiomyocytes with volumes of more than 20000 μm^3^ [5]. In cardiac cell culture of newborn rats, we previously observed the formation of either small immature colony or CICS in the vicinity of large differentiated colony (unpublished observation). In such a case, CICS was produced from the same type of CSC as the adjacent colony. These initial observations were further analyzed using confocal microscopy of freshly isolated myocardial cell suspension obtained from mammals of different age. Figure 4A-4D demonstrates the presence of small poorly differentiated yellow colonies on the surface of large, cardiac protein-positive (red staining) colonies of different maturity. CICSs were identified either on the surface of the mature colonies (Figure 1B and 4E) or inside the colony (Figure 4F) or in the vicinity of the colony (Figure 4G, 4H).

It is well accepted that SCs in general [15], and CSCs in particular [16], are able to undergo either symmetric or asymmetric division. Keeping this in mind, we suggested that asymmetric division of CSC results in the formation of colonies due to sequential divisions of single daughter committed cell. In this case, the other daughter cell retaining the “stemness” state might become activated only after certain time, giving rise to the new colony which is spatially close to the initial colony. It is evident that the secondary colony produced from the same type of CSC as the initial one, will have smaller size and lower level of differentiation, which is shown in Figure 4A-4D. It can not be ruled out, however, that daughter CSC will penetrate one of the cells comprising the colony to form CICS (Figure 4E-4H). Two important suggestions stem from these observations: (i) since the cells of CSC-derived colony are still not fully mature and therefore are smaller than mature CMs, the size of growing CICSs is limited by their dimensions, and (ii) since the colony cells are in fact TACs, which start to differentiate and grow after several rounds of replication, the size of CICSs will increase in parallel to the size of TAC-derived maturating CMs.

**Figure 5:**
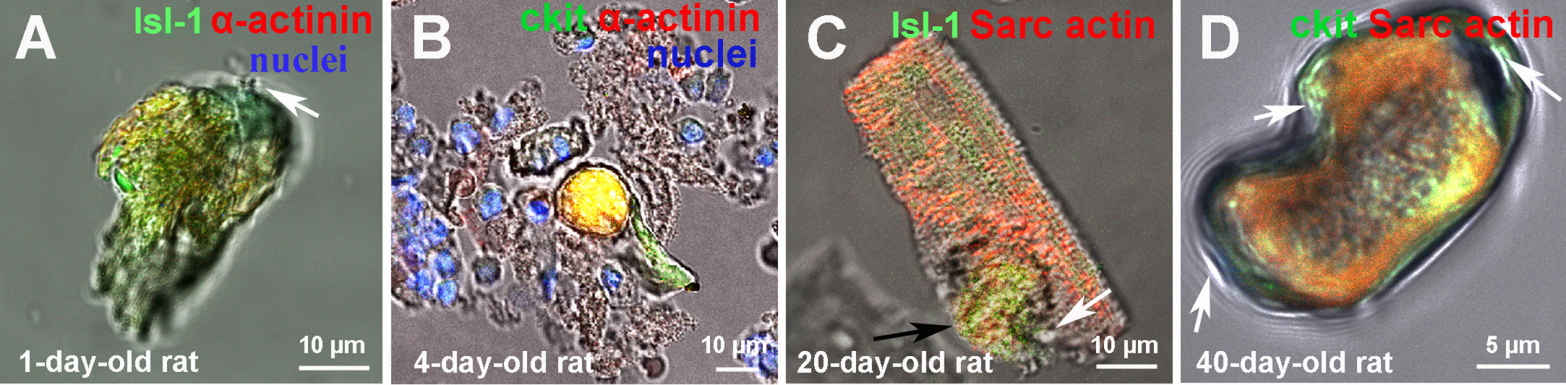
The examples of CSC development inside transitory amplifying cells (TACs) and young cardiomyocytes, revealed in a suspension of ex vivo freshly isolated myocardial cells of rats **(A)** 1-day-old rat Isl-1+ CICS. **(B)** 4-day-old rat c-kit+ CICS. **(C)** 20-day-old rat Isl-1+ CM (reproduced from [14] with permission). **(D)** 40-day-old rat c-kit+ CICS. c-kit+, Isl-1+ (green). Sarcomeric α-actinin, α-Sarcomeric actin (red). Nuclei (Hoechst), blue.

Significant variability of CICS volumes both in *ex vivo* and *in vitro* experiments (Table 1 and 2, respectively) provide strong support for the hypothesis that this variability might be due to differences in the initial volume of host cell and progressive increase in volume during differentiation. Our previous *in vitro* experiments demonstrated that mean volume of cardiac cells during first 8-10 days of culture did not exceed 3246 ± 190 μm^3^ [13]. Table 2 shows that, apart from substantial variability in CICS volume (due to the thickness) there is also a tendency to increase in CICS volume with time. This lends support to the notion that host cell enlargement parallels the growth of CICS. As a result, typical CICS pattern is observed (Figure 3), with encapsulated CSCs occupying almost all space inside host cardiac cell. Moreover, this idea is indirectly confirmed by the fact that CSCs can sometimes penetrate CMs (Figure 5), and not only TACs.

For example, Figure 5A demonstrates the residence of Isl-1^+^ CSC inside young CM from the myocardium of 1-day-old rat. This CM has the length (L) of 41 μm, while its width (W) is 16 μm and 24 μm in the narrow and wide portions, respectively. CSC-containing capsule (L = 27 μm, W = 18 μm, vertical dimension = 9 μm) with a pore on the upper pole (Figure 5A, white arrow) is localized in the upper part of the CM and occupies approximately 30% of CM volume. The capsule with a volume of 3600 μm^3^ was also identified inside the young CM having 40 μm in length in the suspension of myocardial cells obtained from 4-day-old rat (Figure 5B). It is evident that both CICS and surrounding TACs display similar c-kit^+^ phenotype. In turn, mature CM (V = 10000 μm^3^) with Isl-1^+^ bulging (L = 24 μm, W = 19 μm, vertical dimension = 19 μm) was earlier [14] identified in the myocardium of 20-day-old rat (Figure 5C). Despite well-developed sarcomeric structure, expression of α-sarcomeric actin, this large (L = 61.5 μm) CM is Isl-1-positive. The presence of capsule, though without dense wall (Figure 5C, black arrow), evolving pore (Figure 5C, white arrow) and small size are all suggestive of the fact that this “pregnant” CM demonstrates early stage of CSC development inside mature CM. The fourth specific structural pattern of CICSs discriminated on the basis of the analysis of more than 100 CICSs, was identified in the myocardium of 40-day-old rat (Figure 5D). This type of CSC-containing CICS has L = 28 μm, W = 15 μm, and thickness of 38 μm, which forms a capsule with volume of 7800 μm^3^. Relatively small length, unusual shape, even distribution of cardiac (α-Sarcomeric actin) and stem cell (c-kit) marker as well as the presence of 3 openings on both poles and in the constriction area (Figure 5D, arrows) led us to the suggestion that this structure results from invasion of c-kit^+^ CSC in partially committed to a cardiomyocyte lineage TAC. We suppose that despite relatively big initial size, the lack of mature sarcomeric structure allows this TAC to change its morphology, adjusting to the dimensions of invading CSC. More advanced cytoskeleton, seemingly, can interfere with this process leading to formation of such exceptional structure. Besides, since the volume of this structure corresponds well to the volume of average CICS (see Table 1 and 2), it might be suggested that intracellular development of CSCs is taking place not only in early TACs having length ∼10-13 μm and volume < 4000 μm^3^, but also in larger TACs, which are localized at the periphery of the colonies, establishing the contacts with mature CMs, as well as inside young CMs of relatively large size (L = 40-60 μm) [13]. However, this scenario is observed extremely rarely.

**Figure 6:**
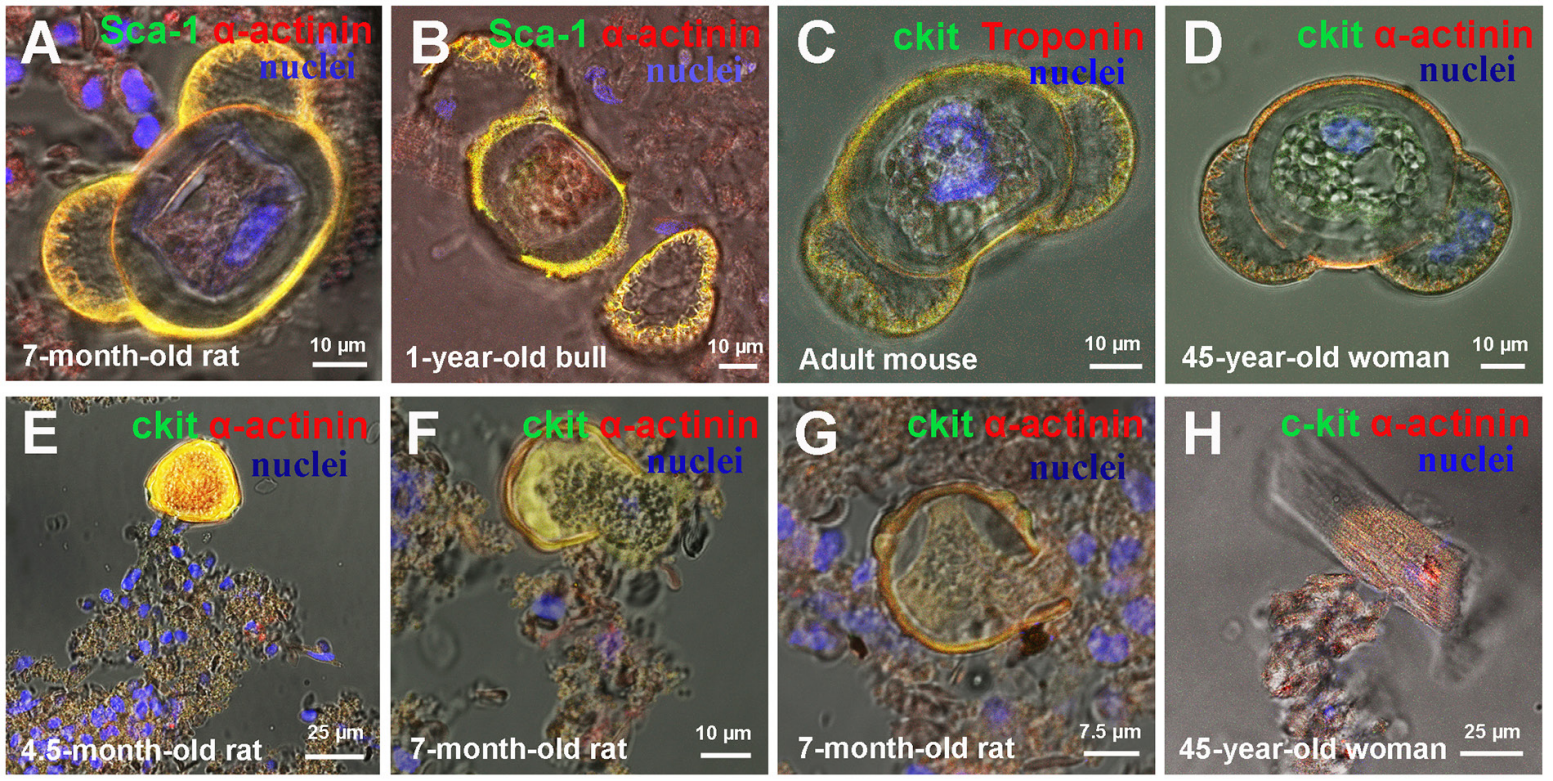
Rupture of cell-in-cell structures (CICSs) and release of transitory amplifying cells (TACs) in mammalian myocardium **(A)** 7-month-old rat, Sca-1+. **(B)** 1-year-old bull, Sca-1+. **(C)** Adult mouse, c-kit+. **(D)** 45-year-old woman, c-kit+. **(E)** 4.5-month-old rat, c-kit+. **(F)** 7-month-old rat, c-kit+. **(G)** 7-month-old rat, c-kit+. **(H)** 45-year-old woman, c-kit+. c-kit+, Isl-1+, Sca-1+ (green). Sarcomeric α-actinin (red). Nuclei (Hoechst), blue.

As earlier in the culture of cardiac cells of newborn, 20-days-old and 40-days-old rats [14], ruptured CICSs and TACs released from them were present in suspension of myocardial cells of mammals of different ages and different species (Figure 6A-6H). It is necessary to emphasize that discovery of TACs in myocardial cell suspension at the stage when TACs are released from the capsule at the vicinity of ruptured CICSs and even at a certain distance from the ruptured CICSs (Figure 6A, 6E-6G) was possible because TACs develop in some mucous media inside CICSs. When released, TACs are submerged into this media and reside there for some time. Similarly, this phenomenon was observed in our previous work, where mucous media and multiple TACs stuck together *in vitro* conditions [14]. Based on the situation shown at Figure 6H when multiple stuck TACs (l=20-30 μm) and a single young CM (l=94 μm) formed from the same type of CSCs (Sca-1^+^), are located in close proximity to each other, we speculate that mucous media likely serves to restrain differentiation of TACs and to create a barrier to hinder penetration of signaling molecules of cardiac differentiation.

**Figure 7:**
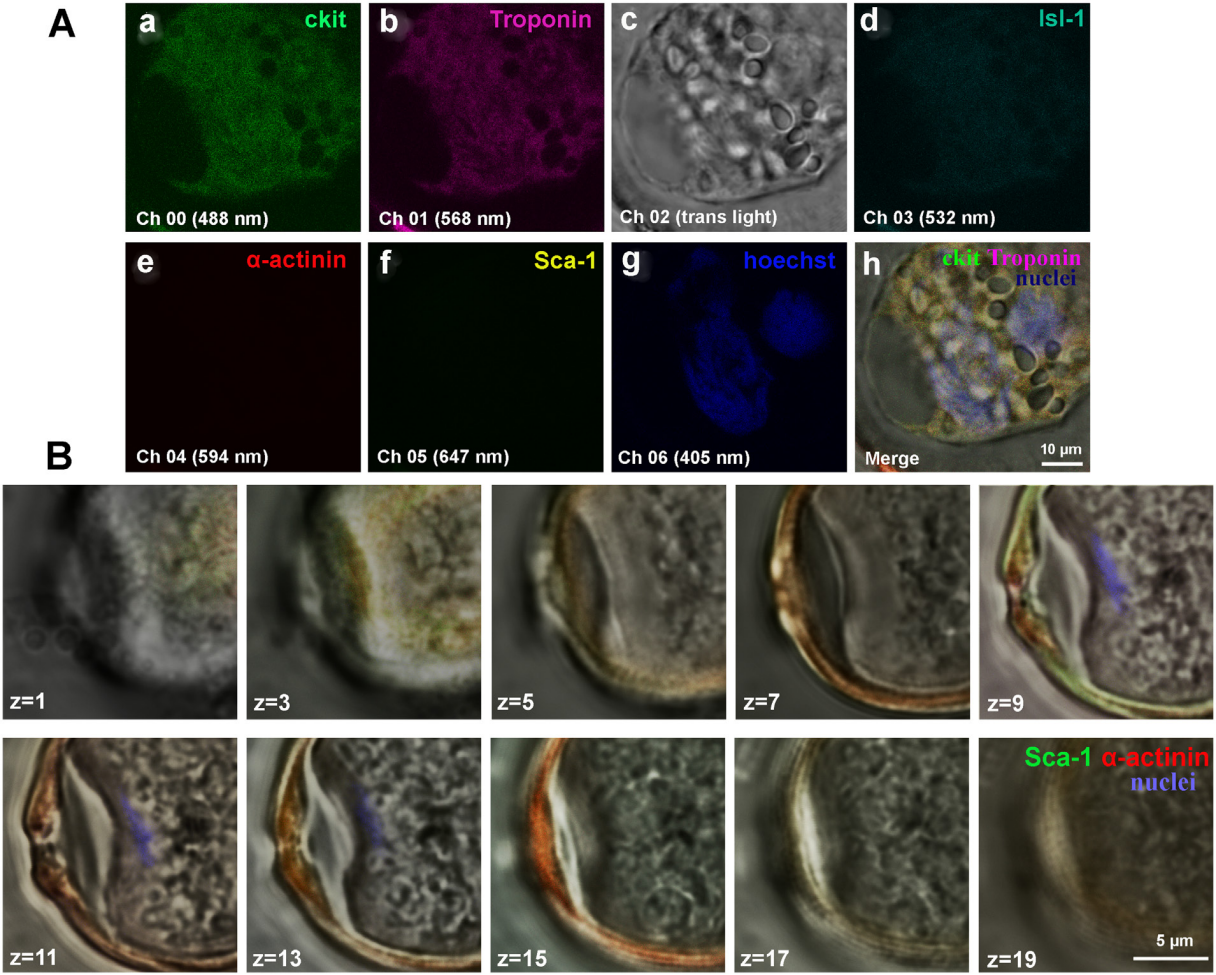
Confocal microscopy of CICSs of one-year-old bull CICSs were stained with antibodies to cardiac stem cell antigens (c-kit+, Isl-1+, Sca-1+) and cardiac proteins (Sarcomeric a-actinin and Troponin T). Hoechst was used for nuclear staining. **(A)** Weak c-kit+ and Troponin expression along with the lack of Isl-1+ and Sca-1+ fluorescence is suggestive of the fact that CICS was produced by the penetration of cardiac myocyte by c-kit+ CSC. **(B)** Z-stacks of Sca-1-positive CICS.

Despite the fact that antibodies against CSC antigens and cardiac proteins specifically bind to corresponding targets within CICSs, we failed to identify discrete cells inside CICSs. We hypothesized that the lack of CSC staining might be explained by intensive absorption of anti-CSC antibodies and Hoechst by highly viscous mucous media surrounding cells inside the vacuole [14]. Here we provide additional evidence supporting this hypothesis. Figure 7A clearly demonstrates diffusion of both antibodies and Hoechst into the vacuole. However, this is not accompanied by individual cell staining. Multiple small round-shaped structures inside the vacuole, presumably cell nuclei, remained colorless. Z-stacks of Sca-1-positive CICS (Figure 7B) support this notion, demonstrating only weak diffuse sarcomeric α-actinin-positive staining of mucous media and local presence of Hoechst in the central part of the vacuole (z = 9-13). Despite the obvious Ki67 diffusion into mucous media as evidenced by intense orange color, it also failed to stain CSC nuclei inside the vacuole (Figure 3A). More detailed information about diffusion of antibodies and Hoechst into CICSs is provided in Figure 3A, 3F, and 3H (see Supplementary Figure 7). It should be noted that clear-cut staining of host cell nuclei by Hoechst shown in Figure 3F-3L is only possible because these nuclei are localized between the capsule wall and the membrane of host cell, thereby making nuclei more accessible for dye.

**Figure 8:**
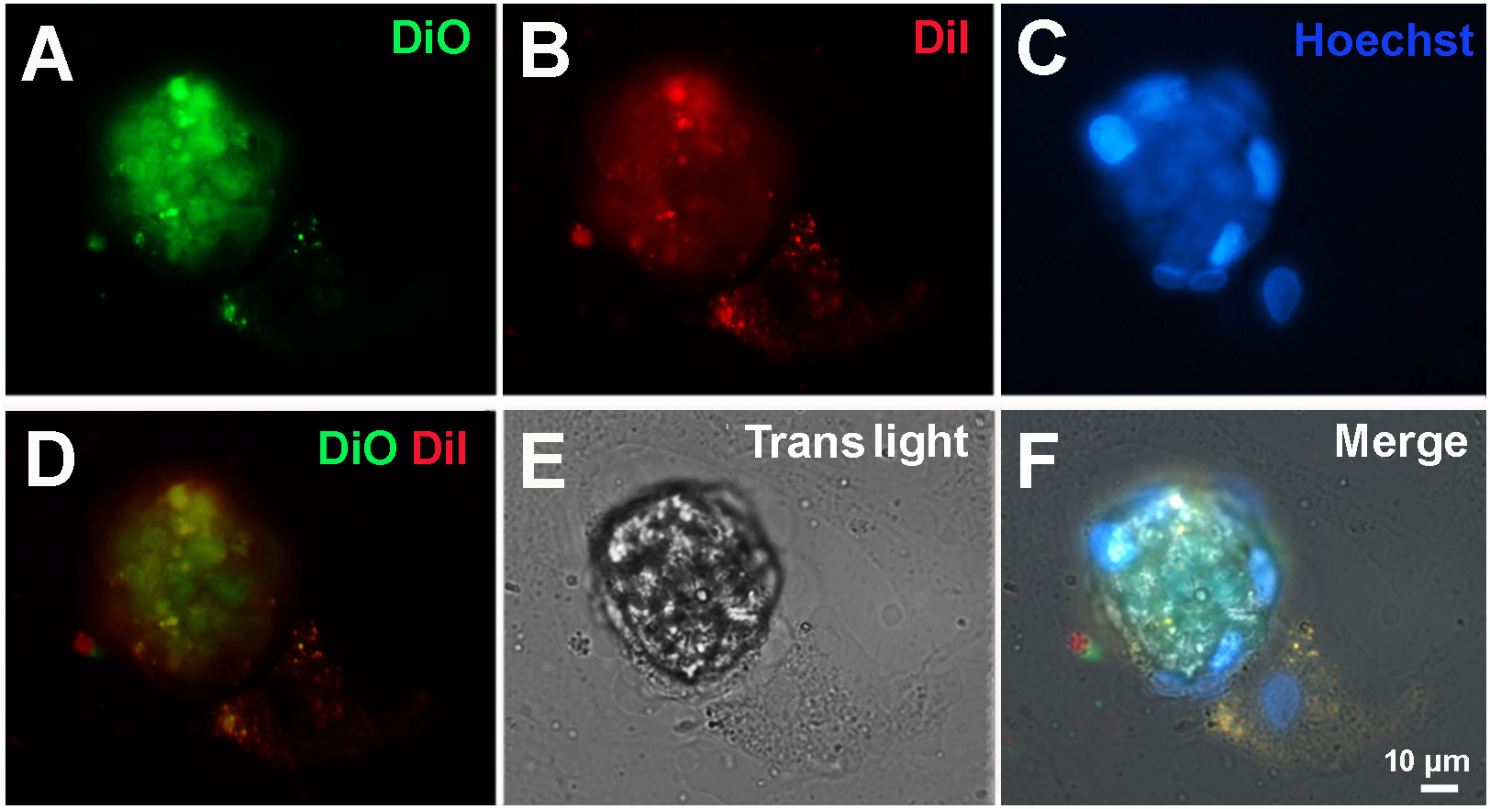
Fluorescent microscopy of CICS formed in the primary cardiac cell culture from 1-day-old rat on the 9th day *in vitro* Cell membranes were stained by 2 vital dyes: DiO (green) and Dil (red). The nuclei were stained by Hoechst (blue).

**Figure 9:**
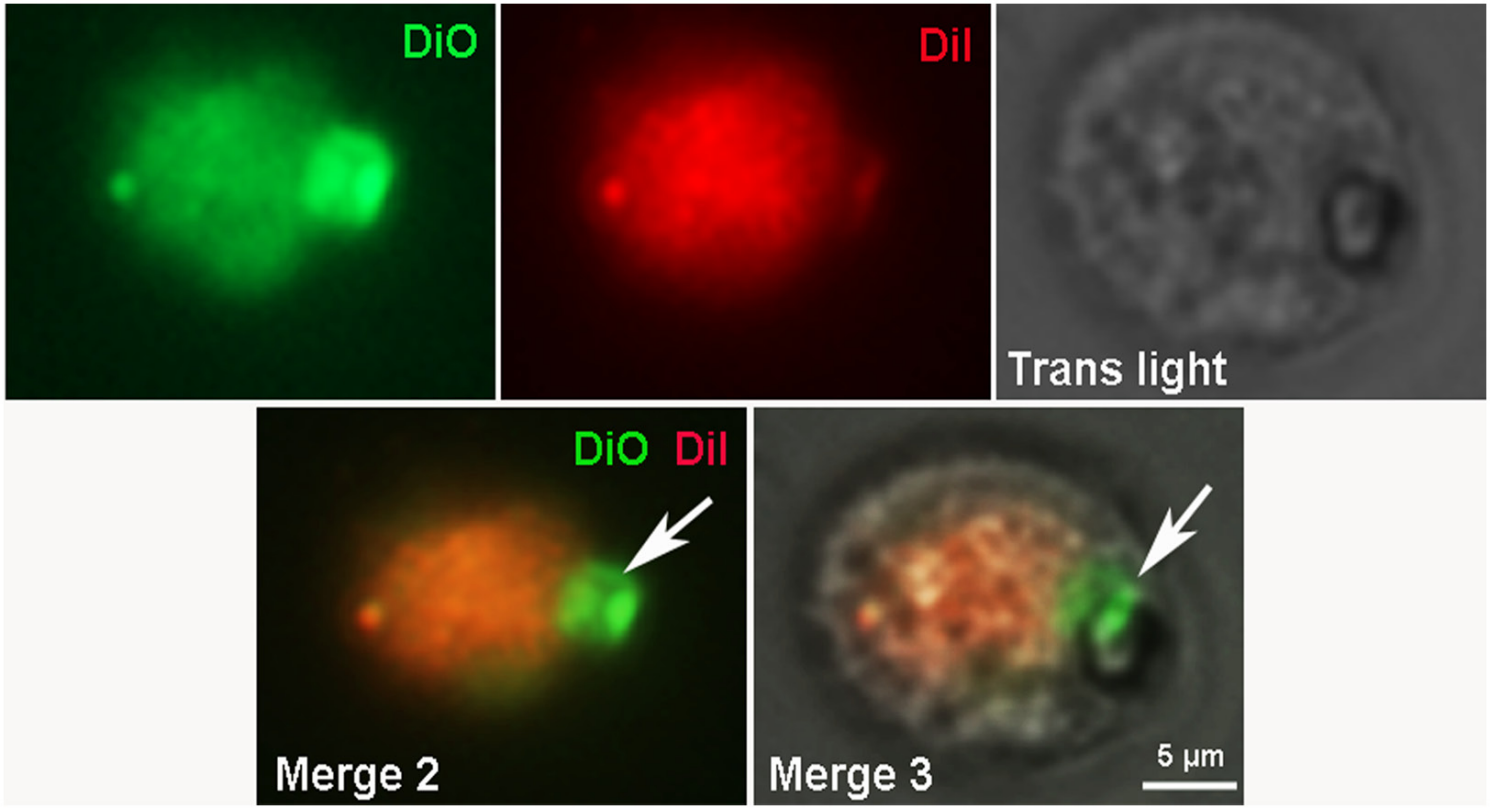
Fluorescent microscopy of CICS formed in the primary cardiac cell culture from 4-day-old rat on the 3rd day *in vitro* Cell membranes were stained by 2 vital dyes: DiO (green) and Dil (red).

**Figure 10:**
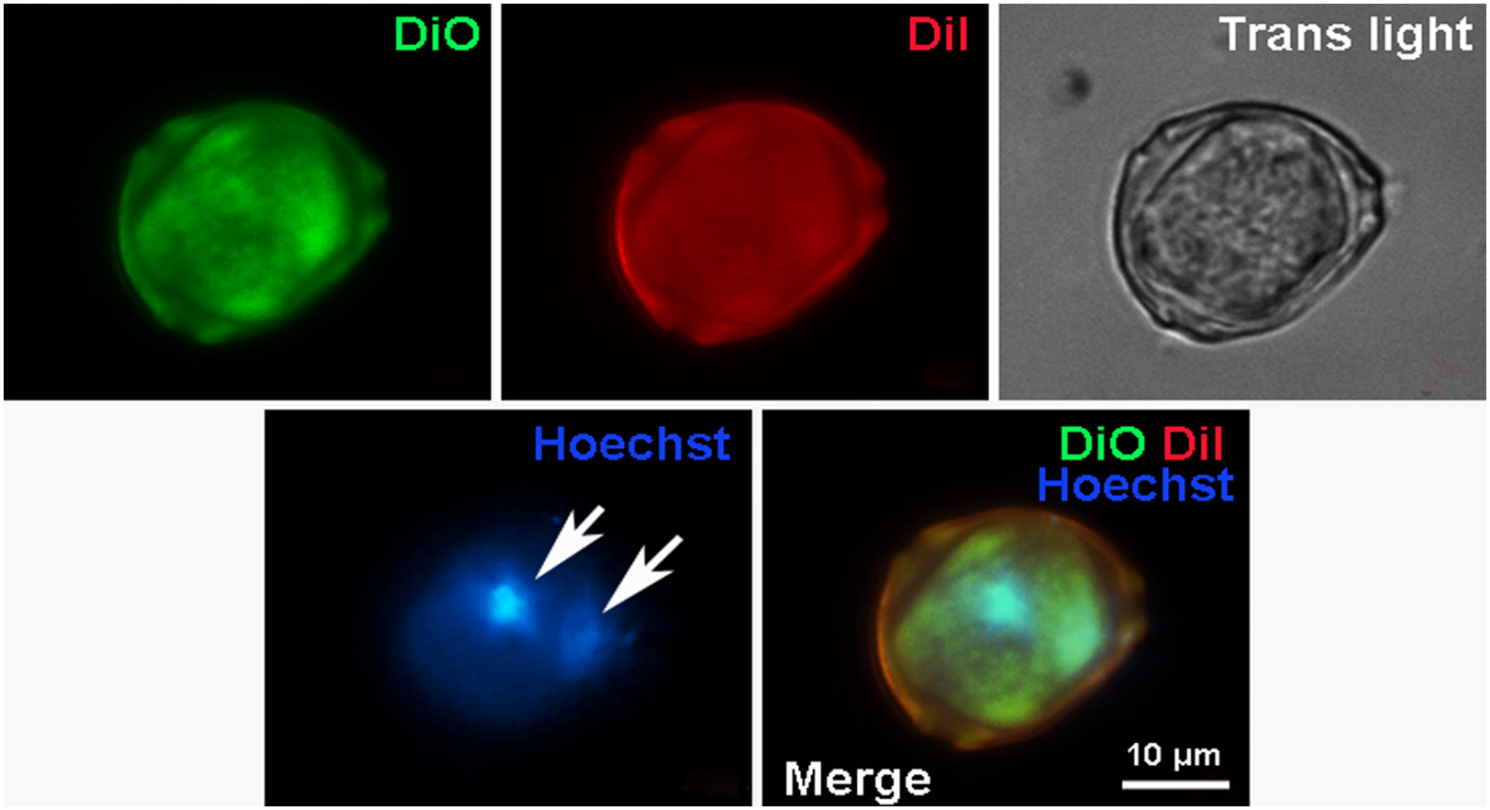
Fluorescent microscopy of mature CICS in the primary cardiac cell culture from newborn rat on the 6th day *in vitro* Cell membranes were stained by 2 vital dyes: DiO (green) and Dil (red). The nuclei were stained by Hoechst (blue).

Additional experiments using two vital dyes (DiO and Dil) complemented by nuclear staining with Hoechst demonstrated that early stages of CICS development are characterized by the presence of small green cells with blue nuclei within the host cell stained red (Figure 8). Merge of figures shown on panels A and B (Figure 8D) clearly indicates that green cells are localized inside the host cell, which is accompanied by pushing away the cytoplasm of host cell to the periphery. Despite the fact that this structure could be visualized much better on Video 5, merging of all images (Figure 8F and Supplementary Video 5) makes it difficult to discriminate between the nuclei of muliple cells within the vacuole and the nucleus(i) of the host cell. Therefore, we performed special experiments with DiO and Dil dyes without accompanying nuclear staining with Hoechst. Using this protocol, we were able to visualize compact aggregation of small red cells inside larger host cell (Figure 9) and on Z-stacks of CICS (Video 6). Despite weak staining of host cell, green thickening on its membrane denotes the area of red cell penetration and further confirms that the membrane of host cell is stained green with DiO. These experiments showed that pre-stained cells were clearly visible inside the host cell at the early stages of CICS development. The open question is whether the cells could be stained inside mature CICS. This issue was explored using both DiO/Dil and Hoechst for nuclear staining (Figure 10). Since DiO and Dil were used simultaneously, both stains marked the outer membrane of the host cell and diffused to the vacuole inside the capsule, while Hoechst readily stained host cell nuclei (arrows). It was also anticipated that the nuclei of cells within vacuole will become Hoechst-positive. However, the membranes and nuclei of the encapsulated cells were not clearly stained in analogy with the experiments on fixed samples stained with antibodies against stem cell and cardiac markers (see Figures 7A and 7B) and Hoechst. Thus, we presume that the lack of CSC staining within the mature CICS could be explained by the accumulation of viscous mucous media inside vacuole which prevents diffusion of antibodies and nuclear stains to their corresponding targets. Moreover, we suggest that mucous media performs two major functions: (*i*) it ensures protection of CSCs within the vacuole from exogenous harmful factors and (*ii*) it may contribute to cardiomyogenic differentiation of CSC progeny (TACs) because it can transport cardiac proteins from the sarcolemmal receptors of host cell, which appeared inside the vacuole during invagination of membrane together with CSC. It is critical to note that within the narrow space of the vacuole TACs acquire only initial cardiac differentiation while retaining the ability to proliferate.

Previously, we showed that CSCs from neonatal rat hearts generated colonies of contracting cardiomyocytes, imitating cardiomyogenesis *in vitro* by passing through several rounds of replication with subsequent differentiation into CMs [13]. This system can be used as an *in vitro* model system of myogenesis to test various compounds and factors, but is not suitable for assessment of the effects of age in proliferation capacities of CSCs and their ability to terminal differentiation in mammals and human.

In this paper we pioneered to propose an *ex vivo* approach to assess activity of resident CSCs in myocardium of mammals by quantification of colonies and CICSs formed by CSCs. This approach helped us to identify CSC-derived colonies of different maturity, determine capacities of CSCs to self-renew myocardium of mammals of different age, and confirmed the phenomenon of intracellular development of resident CSCs in CMs with the formation of CICSs. In the previous work we described the process of CICS formation in primary myocardial cell cultures of newborn, 20-day-old and 40-day-old rats [14]. Here, we confirm the existence of CICSs in adult myocardium from different mammalian species and partly differentiated CSC-derived TACs which are released from ruptured CICSs after several rounds of replication inside the body of CMs.

There is a concept that the potential of myocardium to self-renew and regenerate diminishes throughout life [17] due to the reduced numbers of CSCs, decline in their functional activity [18] or because the sets of CSCs phenotypically become less diverse. Therefore, the role of different subsets of CSCs in colony formation and generation of CICSs in mammals of different age needs a close investigation. In this paper, we studied both processes in different mammalian species, including rat, mouse, steer, and human. In addition, we were interested to investigate whether CICS phenomenon is age-dependent. The third aim of the present work was to evaluate the contribution of c-kit^+^, Sca-1^+^ and Isl-1^+^ CSCs to the formation of colonies and CICSs. In the study, all subsets of CSCs were found in mammals of different age. For example, Sca-1-positive colonies were shown to reside in myocardium of 40-day-old rats (Figure 2A) and a 45-year-old woman (Figure 2I, 2J). In turn, c-kit^+^ colonies were present in adult rats (Figure 2B, 2C), mice (Figure 2D), young bull (Figure 2G) and human (Figure 2H, 2K). At last, Isl-1-positive colonies were defined in myocardium of adult mice (Figure 2E, 2F).

Moreover, we showed that intracellular development with generation of CICSs in myocardium of adult mammals (rats, mice, bull and human) is relevant only for Sca-1^+^ and c-kit^+^ CSCs subsets (See Figure 3). Sca-1^+^ CICSs were present in 4.5-month-old rats (Figure 3B) and 1.5-year-old rats (Figure 3D), as well as in a 1-year-old bull (Figure 3E) and a 45-year-old woman (Figure 3G, 3J-3L). c-kit^+^ CICSs were identified in rats of different age (Figure 3A, 3C), adult mice (Figure 3F) and human (Figure 3H, 3I). It is important to note that Isl-1^+^ CICSs occur in newborn and 20-day-old rats *in vitro* and *ex vivo* [14], but they were not identified in myocardium of older mammals and adult human. The data obtained on rats of different age are summarized in Table 3. We can conclude that c-kit^+^ and Sca-1^+^ CSCs produce both colonies and CICSs, thereby contributing to lifelong myocardial regeneration and self-renewal. However, Isl-1^+^ CSCs seem to be involved in cardiac growth during first month of age only both through colony formation and CICS generation. In turn, the studies on myocardial cell suspensions obtained from adult mice, one-year-old bull and 45-year-old woman not only confirmed the involvement of c-kit^+^ and Sca-1^+^ CSCs in both mechanisms of cardiomyogenesis in mammals of other species, but also showed that Isl-1^+^ colonies are present in the myocardium of adult mice and as a rare event in human (see Table 4).

**Table 3:** Participation of CSCs of various types in the formation of colonies and CICSs in the myocardium of differently aged rats

Type of CSC	Newborn rats		20 day-old rats		40 day-old rats		4.5-month –old rats		7- month –old rats	
	colony	CICS	colony	CICS	colony	CICS	colony	CICS	colony	CICS
c-kit^+^	+	+	+	+	+	+	+	+	+	+
Sca-1^+^	+	+	+	+	+	+	+	+	+	+
Isl-1^+^	+	+	+	+	-	-	-	-	-	-

**Table 4:** Participation of CSCs of various types in the formation of colonies and CICSs in the myocardium of adult mammals

Type of CSC	Adult mouse		1-year-old bull		45-year-old woman	
	colony	CICS	colony	CICS	colony	CICS
c-kit^+^	+	+	+	+	+	+
Sca-1^+^	+	+	+	+	+	+
Isl-1^+^	+	-	-	-	+/-?	-

Our results for the role of c-kit^+^- CSCs in cardiomyogenesis are consistent with data obtained in older rats by Beltrami et al. [19], in old mice and human by Bearzi et al. [20], Hosoda et al. [21], Anversa et al. [22], as well as with the data for Sca-1^+^ CSCs in young [23] and adult mice [24, 25] and human [26]. Despite the multiple facts about the presence of Isl-1^+^ CSCs in early postnatal period of rats and human [27] and in young and old rats [28], their existence in adult mammals and human has long been questioned [29]. In this study, we suggest that formation of mature CMs in adult mice occurs during colony formation from CSCs of different types, including Isl-1^+^ ones.

In addition, capsule rupture of CICSs is accompanied by the release of TACs, which are positive in different extent for the related CSC markers and cardiac proteins. TACs vary in size from 11 μm to 113 μm and have a gradually increasing expression of cardiac proteins which can be indicative of state of differentiation after the release from the ruptured CICSs. Similar morphology (Figure 3) and size of CICSs (Tables 1, 2) in all experimental samples suggests that intracellular development of CSCs in CMs is a common biological event that occurs in mammals of different age and from different species. We assume that together with the formation of CSC-derived colonies, intracellular development of CSCs inside CMs might contribute to self-renewal and, possibly, to myocardial regeneration of mammals.

From the foregoing it follows that rat myocardium of several weeks after birth to very old rats contains CSC-derived colonies of different maturity, as well as ruptured and unruptured CICSs of different size with the released TACs. Importantly, all investigated types of CSCs form colonies and CICSs in young mammals. In contrast, the major role in colony formation and generation of CICS is supposedly played by Sca-1^+^ and c-kit^+^ CSCs in adult and old mammals.

## DISCUSSION

**Figure 11:**
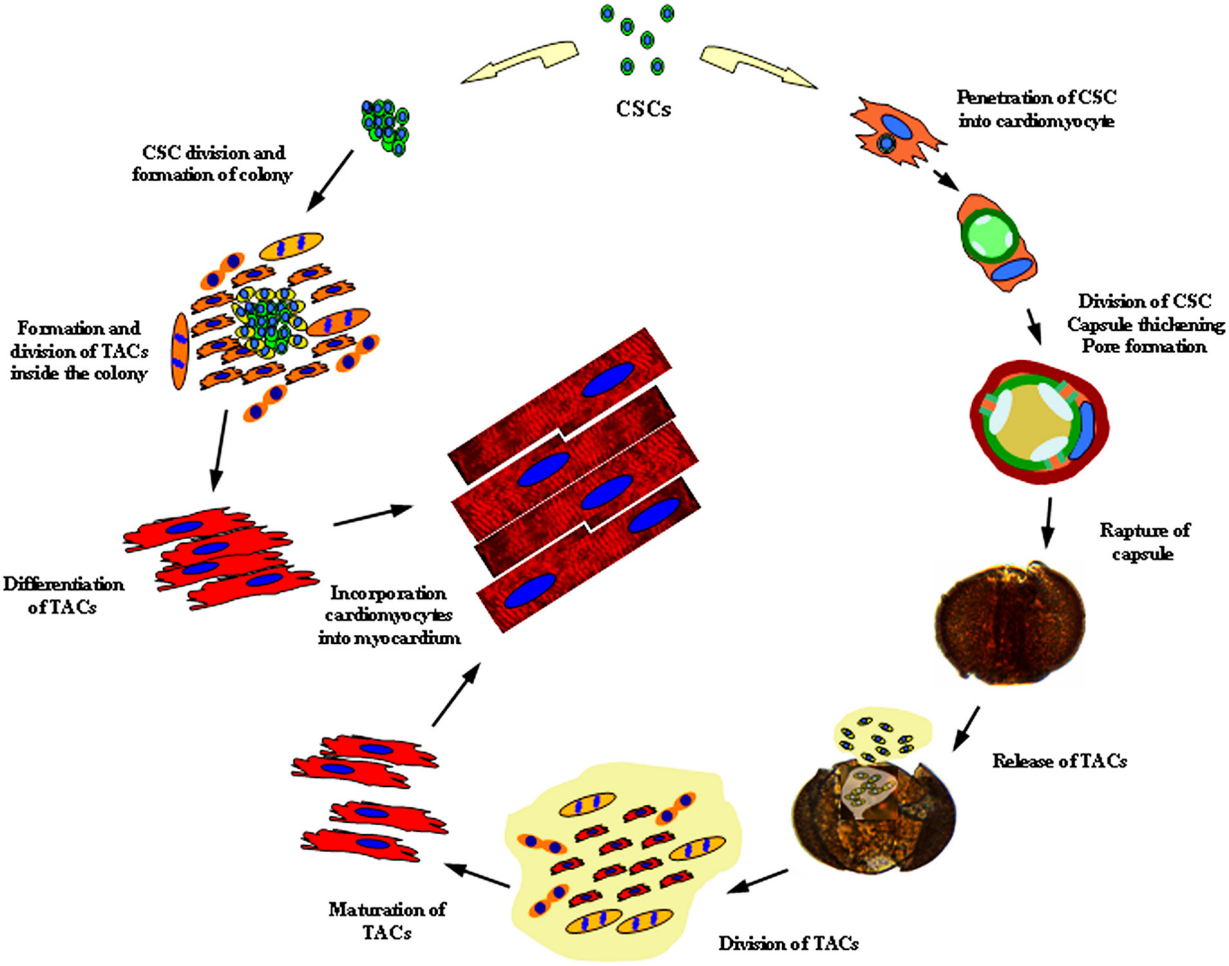
Schematic illustration of two pathways whereby mature CMs are generated from CSCs in mammalian myocardium For details, see text.

Our results suggest that self-renewal of cells in mammalian myocardium is driven primarily by proliferation and differentiation of CSCs inside the colonies and by division and partial differentiation inside the bodies of small myocardial cells (TACs and young CMs). Two processes of mature cardiomyocyte formation are schematically shown in Figure 11. The left side of the proposed scheme reflects the process of colony formation, while the right side depicts the stages of intracellular development of CSCs. We propose that the process of colony formation is associated with several rounds of CSC division, eventually resulting in formation of TACs with different level of differentiation. This occurs only when the colony is formed in contact with mature cells. The latter is a natural event both *in vivo* and *in vitro*, as was shown in our previous paper [13]. The last stage of this process is terminal differentiation of TACs with formation of mature CMs.

The process of intracellular development of CSCs has several distinctive features. First, CSC divides in an enclosed space inside the vacuole, which in turn is surrounded by the capsule inside mature CM. Capsule rupture is followed by the release of TACs partially differentiated into cardiomyocytes, which subsequently terminally differentiate after the mucous media is removed. In contrast to CSC-derived colonies, where TACs differentiate gradually with a part of the cells differentiating soon after division of the primary CSC, while another part continues proliferation, intracellular TACs are believed to have the same initially differentiated phenotype at the stage of their release from CICS which does not prevent further proliferation. As intracellular TACs preserve their capacity to divide, the number of cells capable to terminal differentiation into mature CMs can be increased several folds. As we described previously *in vitro*, ruptured CICS releases as many as ∼200 TACs [14]. Therefore, TACs released from just a single CICS after one or two rounds of cell division and subsequent differentiation can produce more than 400 new CMs.

In this regard it is of interest to consider CSC responses to hypoxia and ischemia. Switching cell culturing conditions the ones that mimic hypoxia and acidosis in injured myocardium helped us to directly interrogate behavior of CSCs similar to that in the infarct zone. For the first time we demonstrated that lowering medium pH to 5 and O_2_ concentration to 85% (hypoxia) blocks differentiation of CSCs inside colonies in cell cultures obtained from newborn rats. However, these deleterious factors stimulated intracellular development of all types of CSCs and enhancing the numbers of CICSs in 5-10 times compared to control.

Given the observation that imitation of ischemia and infarction in *in vitro* experiments suppresses colony formation, but significantly augments intracellular development of CSCs several folds, we suggest that stressful conditions in myocardium and, possibly, in the whole body, can shift the equilibrium between the two processes whereby mature CMs are formed. This suggestion is further supported by the results of our space project Bion-M1. *Ex vivo* investigation of immunocytochemically stained myocardial cells showed that the number of CSC-formed colonies of different maturity was significantly higher in the myocardium of mice subjected to 30-days space microgravity. It should be noted that the number of CICSs was not different from the control animals. Based on this data, we reasoned that weightlessness-induced loss in heart muscle weight is compensated by an increase in the activity of resident CSCs, which form new CMs inside the colonies. However, lack of hypoxia and inflammation in the myocardium during space flight did not stimulate of CSC development inside CMs.

Moreover, our results allow to suggest that passive participation of resident CSCs in regeneration of myocardium may be due to the switch of CSCs to the intracellular path of development when exposed to inflammation, rapid fibrosis, and circulatory failure in myocardial infarction [30]. It is plausible that stressful conditions force CSCs to hide inside mature CMs. However, even under optimal conditions, development of CSCs inside CMs with the following generation of CICSs is 2-2.5 times longer in time compared to the formation of differentiated CMs inside the colonies: 20-25 days vs 10-11 days, respectively. Therefore, suppression of colony formation and a switch of CSCs to intracellular path of development during ischemia and infarction can substantially reduce or even completely rule out the role of CSCs in myocardium regeneration in an acute phase of disease. It is fairly possible that stressful conditions nullify the effects of endogenous SCs as well as of exogenous SCs, including pluripotent SCs and iPSCs, injected into damaged myocardium [31]. However, contribution of TACs released from CICSs in regenerative processes at later stages post-infarction and in chronic heart failure can be essential.

In the context of our hypothesis, it is necessary to discuss another crucial question on the agenda of cardiology: what kind of cells (resident CSCs or mature CMs) renew adult myocardium? Recent papers where myocardium of mammals was found to contain small cardiac-positive cells with an ability to divide are of particular interest. For instance, Omatsu-Kanbe et al. [7, 32] described a population of small cells (∼10-18 μm) in myocardium of adult mice, differentiating into contractile CMs without prior division in *in vitro* conditions. In turn, Kimura et al. [8] showed at mice myocardium that hypoxia induces formation of small dividing cells with characteristics similar to that of neonatal CMs, i.e. a small size (∼10 μm), mononuclearity and minor oxidative DNA damages. Based on this data, the authors suggest that there are neonatal-like CMs in myocardium of adult mammals with a capacity to proliferate not only at the first days after birth, but through the life-time. Moreover, recent data from Tang et al. [9] prove that 90-minutes ischemia with the following reperfusion and injection of allogenic c-kit^+^ CSCs stimulates a prolonged proliferative response of endogenous a-Sarcomeric actin-positive cells that do not have a phenotype of mature CMs. Considering the fact that division of large (>20000 μm^3^) and terminally differentiated CMs is doubtful or even denied [5], and small dividing cells exceed resident CSCs in size, the source and physiological role of these cells remains obscure.

Our results overarch recent data and point to the possibility that TACs released from the intracellular capsule of CICSs in our studies represent CMs of the «new type» described by Omatsu-Kanbe, et al. [7, 32] as well as small immature neonatal-like CMs, produced in hypoxia [8] and ischemia [9]. In the former paper [7] the authors transferred the supernatant obtained by centrifugation of a suspension of adult mice myocardial cells (light fraction) into cell culture. To our understanding, this fraction always contains TACs released after the rupture of mature CICSs. In the paper by Kimura et al. [8] neonatal-like CMs produced in hypoxic conditions are very similar to cells obtained during stressful conditions stimulating the intracellular development of CSCs in our *in vitro* studies. In addition to that, our data on CSCs hiding inside mature CMs under stressful conditions suggest that aSA-positive small cells observed by Tang et al. [9] after occlusion of the aorta could have been transitory amplifying cells released from CICSs, as shown in our experiments.

Because of none of the abovementioned papers consider small neonatal-like CMs to be derived from a stem cell source, it is important to underscore that expression of stem cells antigens gradually declines during intracellular development in the descendants of the parental CSC. At the same time, synthesis of cardiac proteins (a-sarcomeric actin, sarcomeric a-actinin, troponin T) increases. This particular observation makes them attributable neither to CSCs, nor to a population of mature cardiomyocytes. TACs have a transitory phenotype, characterized by a larger size compared to CSCs (10-18 μm vs 5-6 μm), varying levels of cardiac protein expression, weak or extremely weak properties of stem cells, but a preserved ability to divide. Not surprisingly, the source of small dividing cardiac-positive cells has remained unexplained out of the context of intracellular development phenomenon. In addition, proliferative capacities of TACs can be considered as the primary source of regeneration in myocardium of adult mammals. We hypothesize that these cells can not only support homeostasis of the heart by executing the processes of self-renewal throughout the whole life of the organism, but also to partially contribute to cardiac regeneration in post-infarct stage of disease or in chronic heart failure. This assumption is in good agreement with the view of Malliaras et al. [33], which using an inducible genetic labeling approach identified small (11.5±3.7 μm) nonmyocyte cells expressing cardiac markers which they named cardioblasts. Besides, the authors have found that endogenous cardioblasts are rarely observed in the normal adult mouse heart, whereas their number is becoming tenfold increased after myocardial infarction. In addition, genetically labelled cardioblasts were shown to express cardiac transcription factors and sarcomeric proteins, demonstrated spontaneous contractions and gave rise to mature CMs after intramyocardial injection *in vivo*. It was unequivocally shown that these “endogenous cardioblasts” do not arise from hematogenous seeding, cardiomyocyte dedifferentiation, or just expansion of a preformed progenitor pool [33].

It is surprising that despite the availability of data on the residence of small proliferating cells with cardiomyogenic potential in the adult heart, many authors [34–37], including those who described “endogenous cardioblasts” [33], continue to believe that proliferation of pre-existing cardiomyocytes is the dominant mechanism for generation of CMs in adult mammalian myocardium. The ignorance of sharp contrast between the sizes of proliferating cells and mature CMs results in futile attempts to develop various ways of stimulating proliferation of pre-existing cardiomyocytes. It is clear that the impact of TACs and other types of cardiac-positive small cells (neonatal-like CMs or cardioblasts) on the heart in acute myocardial infarction needs to be determined. Moreover, precise investigation of their role in heart homeostasis of mammals will pave the new way for cell-based tools in regenerative medicine.

## MATERIALS AND METHODS

### Animals

Wistar rats of different ages, 19-20–week-old C57/bl6N mice and one-year-old bull were used throughout the study. The experiments were performed in accordance with the Guide for the Care and Use of Laboratory Animals and were approved by a local Ethics Committee.

### Mice

The experiments were approved by the local Ethics Committee at the Institute of Mitoengineering, Moscow State University and the Institute of Medical Biological Problems, Russian Academy of Sciences. Male C57/bl6N mice of SPF category were purchased from animal breeding facility at Shemyakin-Ovchinnikov Institute of Bioorganic Chemistry (Pushchino, Russian Federation). The animals were maintained on a 12 h light/dark cycle and were provided food and water *ad libitum*. The animals were euthanized by cervical dislocation performed by the experienced researcher.

### Rats

The animals were euthanized by asphyxiation in CO_2_ chamber, after which organ samples were taken.

### Cattle

Myocardial sample has been taken from 1-year-old steer at the farm immediately after killing.

### Human

A fragment of human myocardium was isolated from a 45-year old woman post mortem, who died not due to cardiac disease or cardiac injury. Bioptic specimen was taken after the written informed consent was obtained from patient's relatives. The investigation conformed to the principles outlined in the Helsinki Declaration and was granted by the local ethics committee.

### Isolation of cardiac cells for *ex vivo* experiments

Dissected hearts or myocardial fragments were enzymatically dissociated into a single cell and small fragment suspension as previously described [14]. Briefly, the hearts were excised and rinsed in Ringer’s solution (pH 7.4) consisting of 146 mM NaCl, 5 mM KCl, 2 mM CaCl2, 1 mM MgCl2, 11 mM glucose, and 10 mM HEPES. After mincing and incubation in the same solution with the addition of 1 mg/mL collagenase IA (Sigma-Aldrich, USA) and 0.12% trypsin (FLUKA, Sigma-Aldrich) at 37°C for 30 min, the suspensions thus obtained were left to rest without further stirring for 2–3 min to precipitate the undissociated tissue fragments. The supernatant was centrifuged at 400 × g for 10 min for the enrichment of viable cells. Gentle pipetting and centrifugation were used to preserve the integrity of myocardial CSC-derived colonies and CICSs. The enzyme-free cell suspension was stained using antibodies, followed by suspending the cells between the slide and cover slip.

This particular technique of cell isolation was used because we aimed to maintain the integrity of specific cellular structures, including CSC-derived colonies and CICSs, which are not identifiable with use of other approaches. Given rare occurrence of CSC-derived colonies and CICSs (1 colony or CICS per 100 000 CMs), we were as yet unable to identify and describe these structures using FACS analysis, confocal or electron microscopy of myocardial specimens. In this regard, the presented technique of *ex vivo* characterization of freshly isolated mammalian myocardial cells seems to be optimal for identification of previously unknown CICSs and CSC-derived colonies of different size and maturity,

### Immunocytochemistry

After rinsing with PBS and fixation for 20 min in 2.5% paraformaldehyde at room temperature, the cells were permeabilized with 0.25% Triton-X100 for 10 min. For immunostaining we used primary mouse anti-Isl-1 antibodies (Abcam) and mouse antibodies to sarcomeric alpha-actinin (Abcam), alpha-sarcomeric actin (Sigma-Aldrich) and cardiac troponin T (Abcam) pre-conjugated with Alexa 532, 546, 568, 594, or 647 according to Zenon technology (Invitrogen). Phycoerythrin-conjugated anti-Ki-67 antibodies were used for determination of proliferative capacity of CSCs. Commercially available FITC-conjugated anti-c-kit (Abcam) and anti-Sca-1 (Abcam) antibodies were also used at 1:100 dilution. Hoechst 33342 (10 μg/mL, Molecular Probes, USA) staining at 1:1000 dilution was used for detection of cell nuclei.

Confocal microscope Leica TCS SP5 provides an opportunity to simultaneously analyze several fluorescent tags in wide spectrum (from ultraviolet to infrared), thus it is possible to use several fluorochromes and visualize expression of CSCs markers of several types at a single sample. Intensity of the corresponding fluorochrome serves to establish a type of CSC which generated a colony or CICS.

### Experiments with lipophilic membrane fluorescent dyes Dil and DiO

Fluorescent membrane dyes Dil (Ex/Em - 549/565 nm, Molecular Probes, Eugene, USA) and DiO (Ex/Em - 484/501 nm) were used to stain the membranes of living myocardial cells in red and green color, respectively. For this purpose, freshly isolated suspension of cardiac cells from newborn rats was divided into two fractions, one of which was incubated with DiO and another - with Dil for 20 min at 37°C. After that, the cells were precipitated at 400 g for 10 min with subsequent washout from fluorophores. This was followed by combination of both fractions in warm DMEM supplemented with 10% fetal calf serum (Biovitrum, Russia), 50 U/ml of penicillin, and 50 μg/ml of streptomycin (Biolot, Russia). The cells were incubated with Hoechst 33258 (Ex/Em – 352/461 nm) at concentration of 3 μM for 15 min under room temperature. This approach allowed to visualize both host cell and CSC nuclei. After removal of Hoechst, Dil/DiO-stained cells were plated onto the glass bottom dishes with collagen coating (MatTek Corporation) and incubated at 37°C according to previously reported protocol [13].

### Visualization

A confocal microscope (Leica TCS SP5, Germany) with 20× and 63× glycerol objectives was used to visualize cells. For optical tomography, the sections were spaced 1.01 μm along the z-axis. Live myocardial cells stained with DiO, Dil, and Hoechst were visualized using inverted fluorescent microscope (Axio Observer.Z1, Carl Zeiss, Germany).

### Cytometry

Linear cell dimensions were obtained using commercially avaliable software for confocal microscope (Leica TCS SP5) and were used for calculation of cell volume (V) using the following formula: V = 3.14 / 6 × L × W ×vertical dimension.

### Statistical analysis

All of the data are expressed as the mean ± standard deviation. The statistical analyses were performed using the SPSS 13.0 software package (SPSS Inc. Software, USA).

## SUPPLEMENTARY MATERIALS FIGURES AND VIDEOS















## Abbreviations

CICSs: cell-in-cell structures
CMs: cardiomyocytes
CSCs: cardiac stem cells
TACs: transitory amplifying cells
