# Supplementary material for: Two mechanisms of cardiac stem cell-mediated cardiomyogenesis in the adult mammalian heart include formation of colonies and cell-in-cell structures
Source: Oncotarget. 2018 Sep 25;9(75):34159–75. doi: 10.18632/oncotarget.26148 (PMC6183336; doi:10.18632/oncotarget.26148)
Supplement: Supplementary file 1 [file oncotarget-09-34159-s001.pdf]

Two mechanisms of cardiac stem cell-mediated cardiomyogenesis in the adult mammalian heart include formation of colonies and cell-in-cell structures

SUPPLEMENTARY MATERIALS

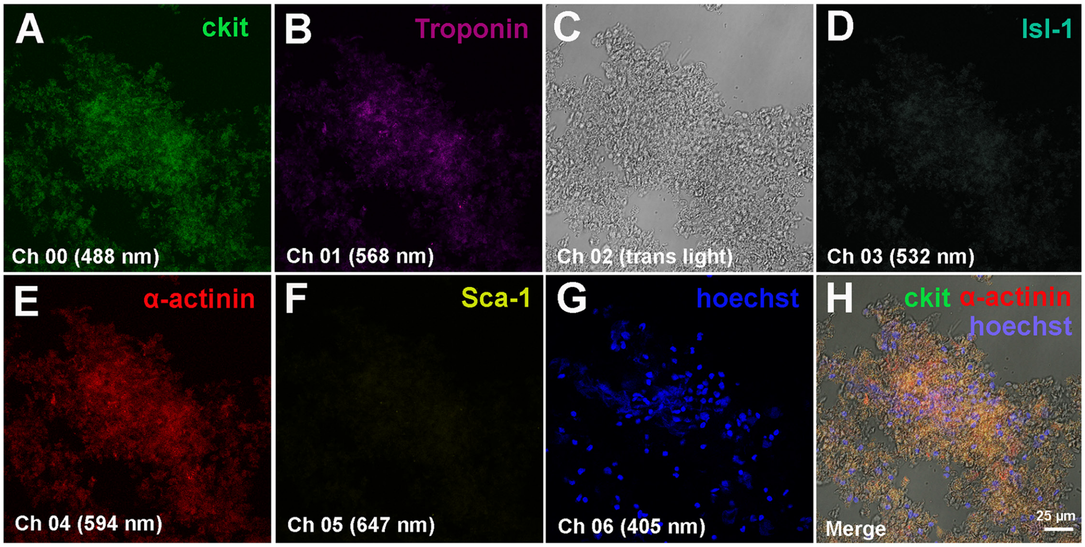

Supplementary Figure 1: Expression of CSC and cardiac markers of several types with simultaneous use of several fluorescent tags as shown on Figure 2B. Confocal microscopy of c-kit+ colony obtained from 7-month-old rat. **(A)** c-kit+. **(B)** Troponin. **(C)** Transmitted light. **(D)** Isl-1+. **(E)** Sarcomeric α-actinin. **(F)** Sca-1+. **(G)** Hoechst (nuclei). **(H)** Merged c-kit+, Sarcomeric α-actinin, and Hoechst staining on transmitted light image.

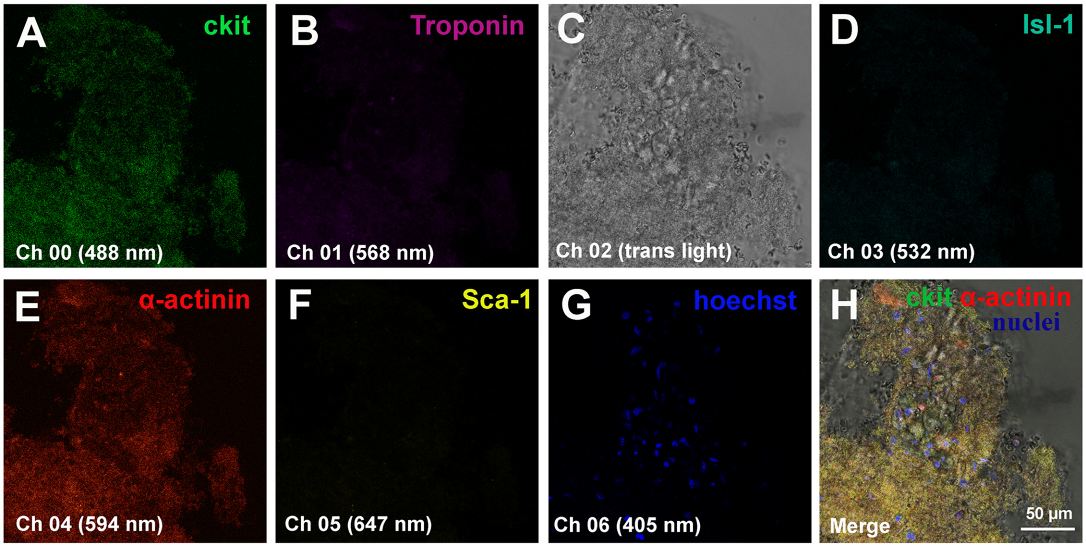

Supplementary Figure 2: Expression of CSC and cardiac markers of several types with simultaneous use of several fluorescent tags as shown on Figure 2C. Confocal microscopy of c-kit+ colony obtained from 1.5-year-old rat. **(A)** c-kit+. **(B)** Troponin. **(C)** Transmitted light. **(D)** Isl-1+. **(E)** Sarcomeric α-actinin. **(F)** Sca-1+. **(G)** Hoechst (nuclei). **(H)** Merged c-kit+, Sarcomeric α-actinin, and Hoechst staining on transmitted light image.

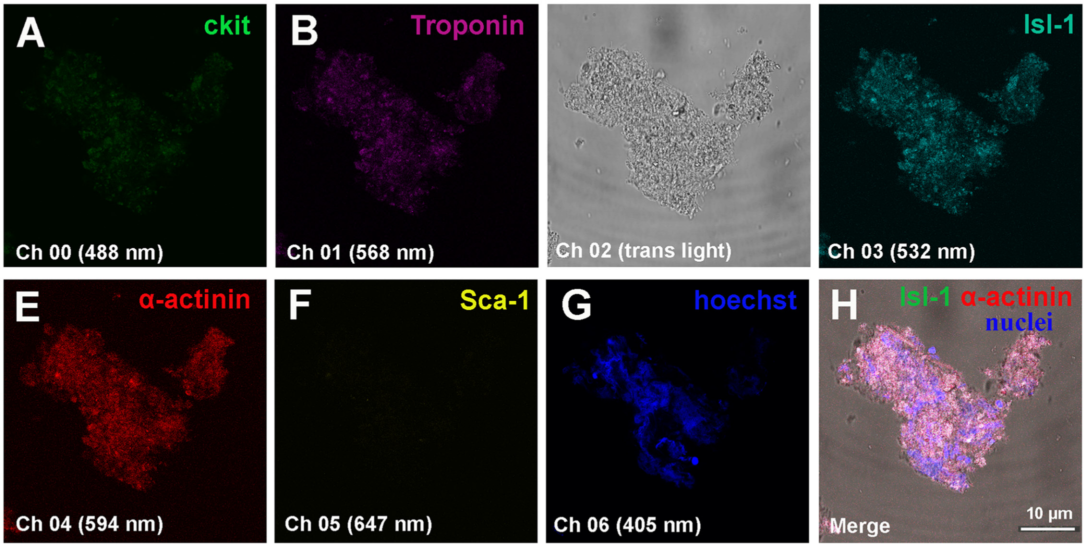

Supplementary Figure 3: Expression of CSC and cardiac markers of several types with simultaneous use of several fluorescent tags as shown on Figure 2F. Confocal microscopy of Isl-1+ colony obtained from adult mouse. **(A)** c-kit+. **(B)** Troponin. **(C)** Transmitted light. **(D)** Isl-1+. **(E)** Sarcomeric α-actinin. **(F)** Sca-1+. **(G)** Hoechst (nuclei). **(H)** Merged Isl-1+, Sarcomeric α-actinin, and Hoechst staining on transmitted light image.

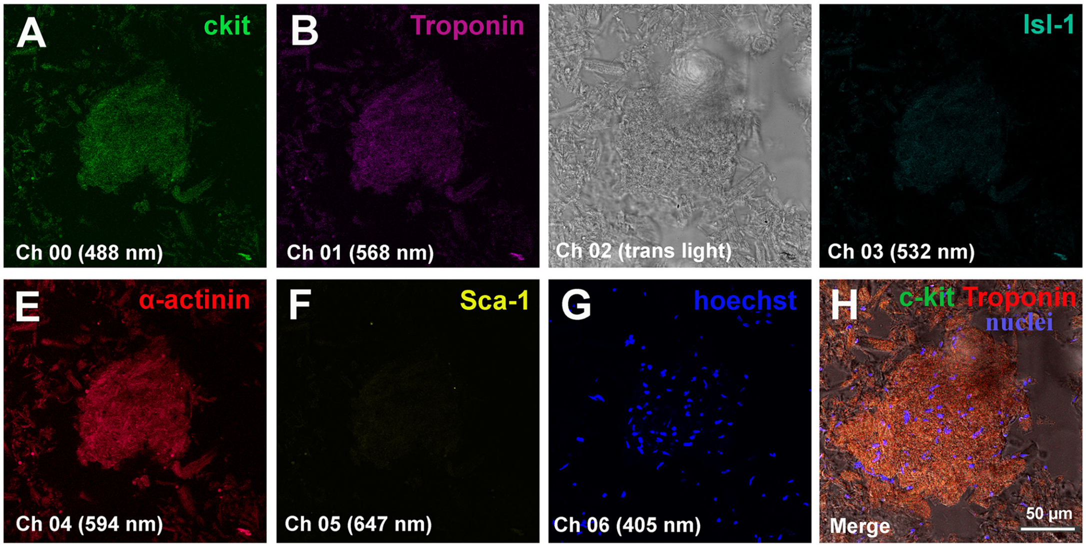

Supplementary Figure 4: Expression of CSC and cardiac markers of several types with simultaneous use of several fluorescent tags as shown on Figure 2G. Confocal microscopy of c-kit+ colony obtained from one-year-old bull. **(A)** c-kit+. **(B)** Troponin. **(C)** Transmitted light. **(D)** Isl-1+. **(E)** Sarcomeric α-actinin. **(F)** Sca-1+. **(G)** Hoechst (nuclei). **(H)** Merged c-kit+, Sarcomeric α-actinin, and Hoechst staining on transmitted light image.

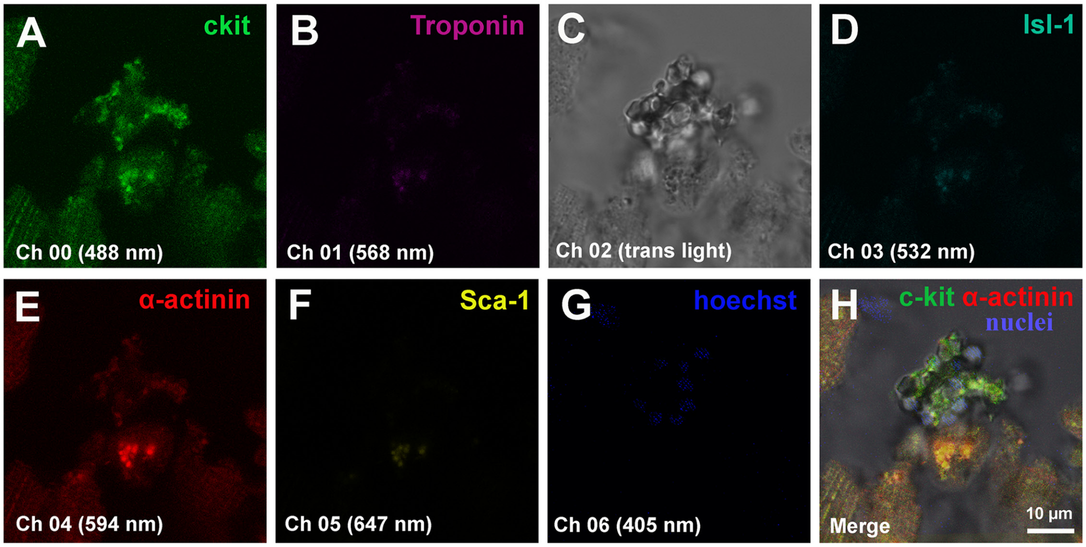

Supplementary Figure 5: Expression of CSC and cardiac markers of several types with simultaneous use of several fluorescent tags as shown on Figure 2H. Confocal microscopy of c-kit+ colony obtained from 45-year-old woman. **(A)** c-kit+. **(B)** Troponin. **(C)** Transmitted light. **(D)** Isl-1+. **(E)** Sarcomeric α-actinin. **(F)** Sca-1+. **(G)** Hoechst (nuclei). **(H)** Merged c-kit+, Sarcomeric α-actinin, and Hoechst staining on transmitted light image.

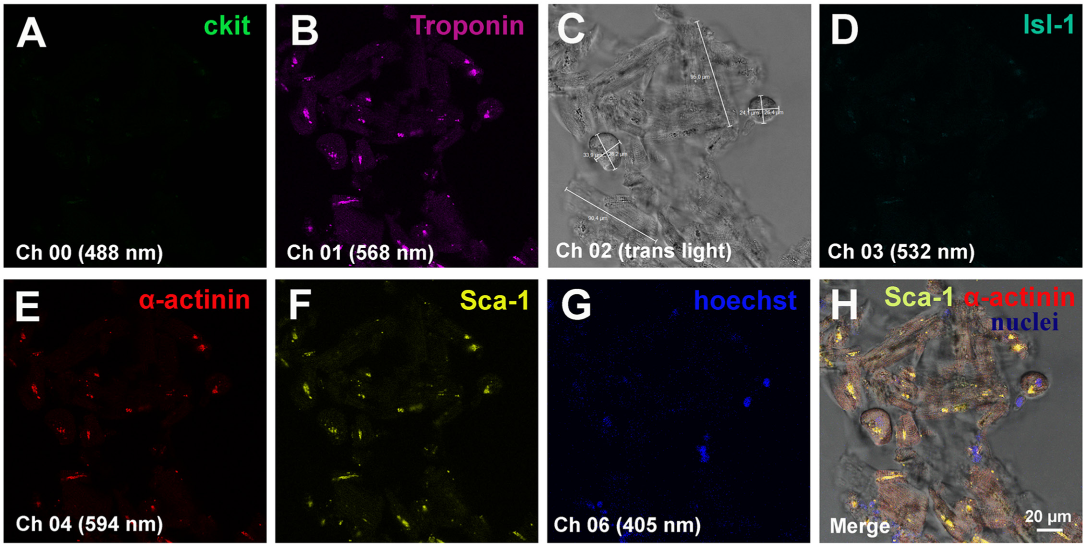

Supplementary Figure 6: Expression of CSC and cardiac markers of several types with simultaneous use of several fluorescent tags as shown on Figure 2I. Confocal microscopy of Sca-1+ colony obtained from 45-year-old woman. **(A)** c-kit+. **(B)** Troponin. **(C)** Transmitted light. **(D)** Isl-1+. **(E)** Sarcomeric α-actinin. **(F)** Sca-1+. **(G)** Hoechst (nuclei). **(H)** Merged Sca-1+, Sarcomeric α-actinin, and Hoechst staining on transmitted light image.

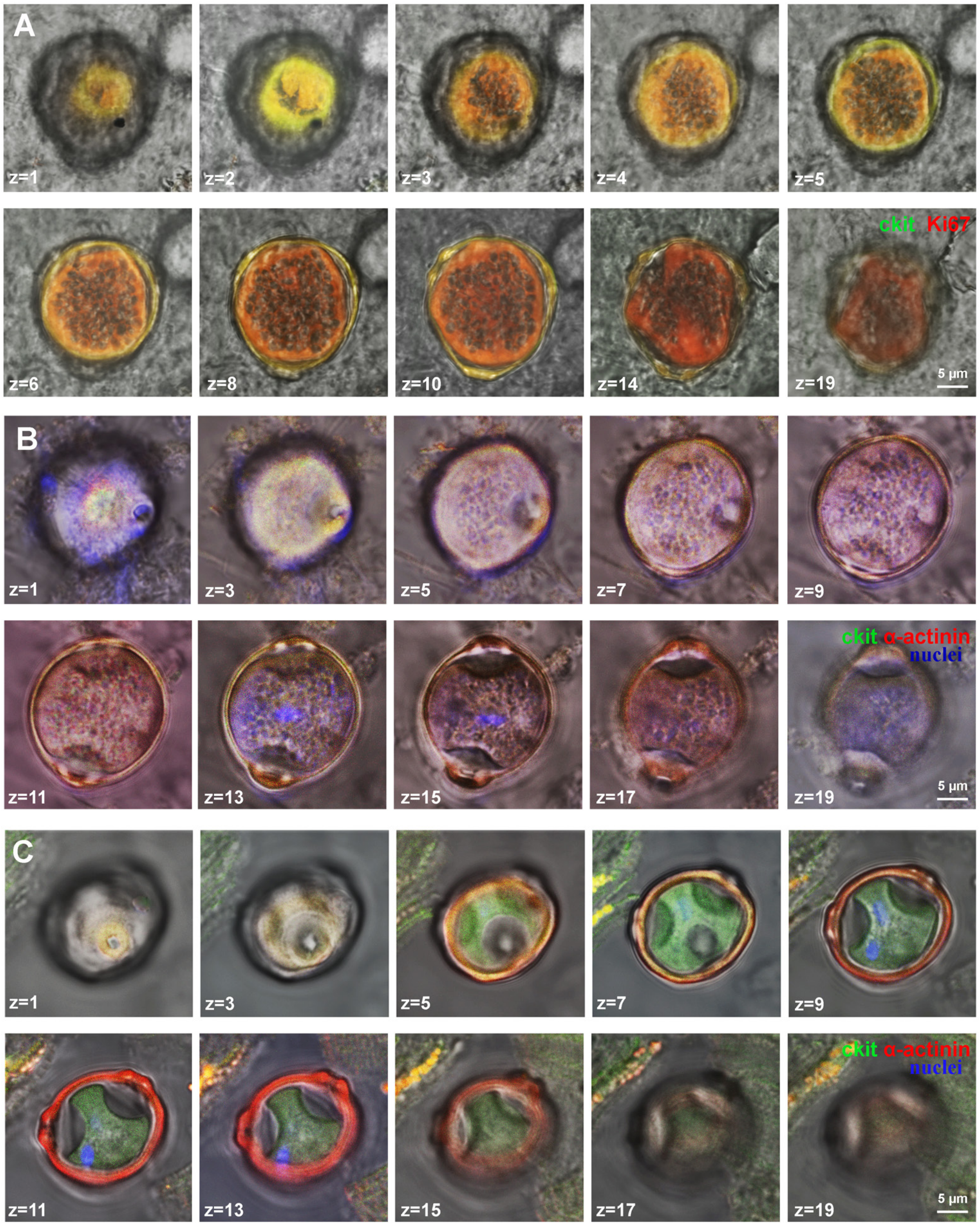

Supplementary Figure 7: Z-stacks of merged c-kit+, Sarcomeric α-actinin, and Hoechst staining on transmitted light images corresponding to specific panels of Figure 3. **(A)** Figure 3A, 40-day-old rat. **(B)** Figure 3F, adult mouse. **(C)** Figure 3H, 45-year-old woman.

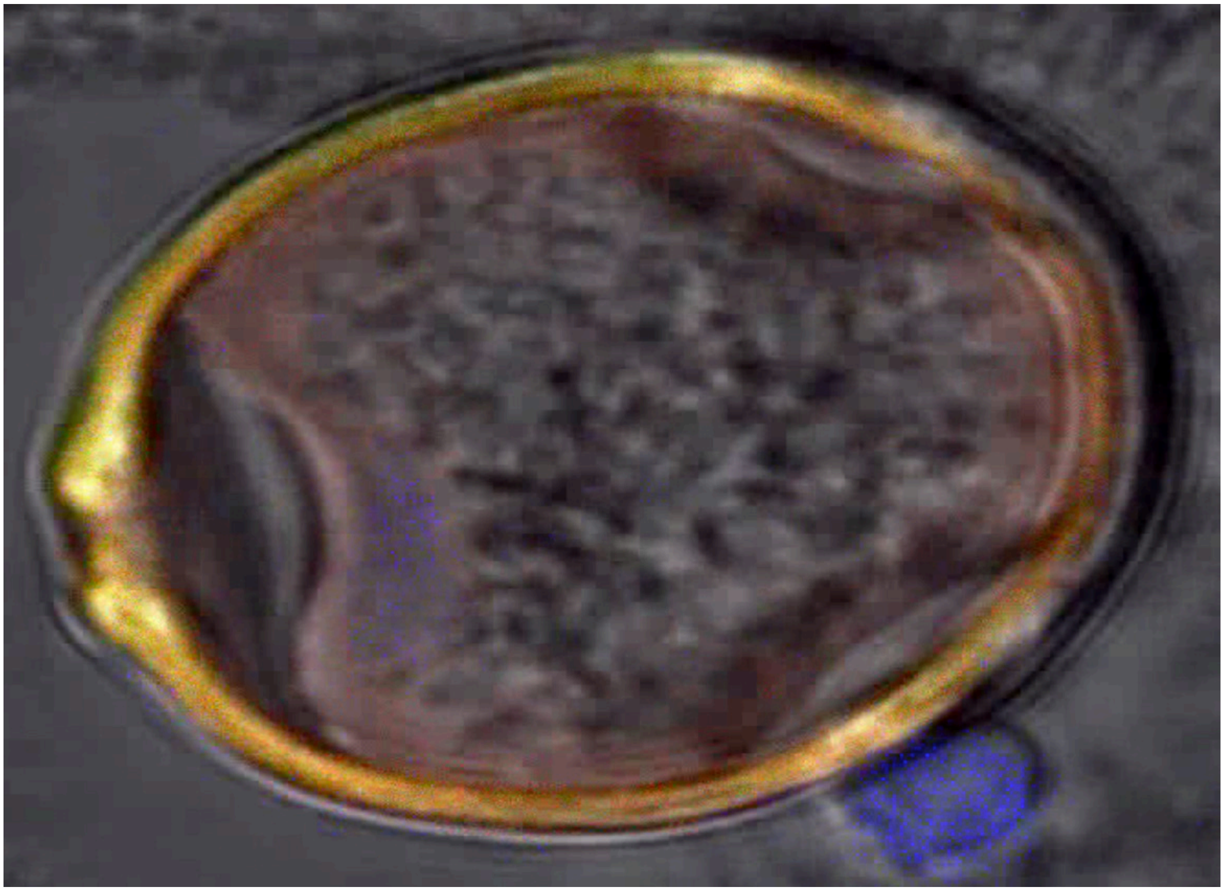

Supplementary Video 1: Scanning of a CICS with 3 pores from the heart of an one-year-old steer.

See Supplementary File 1

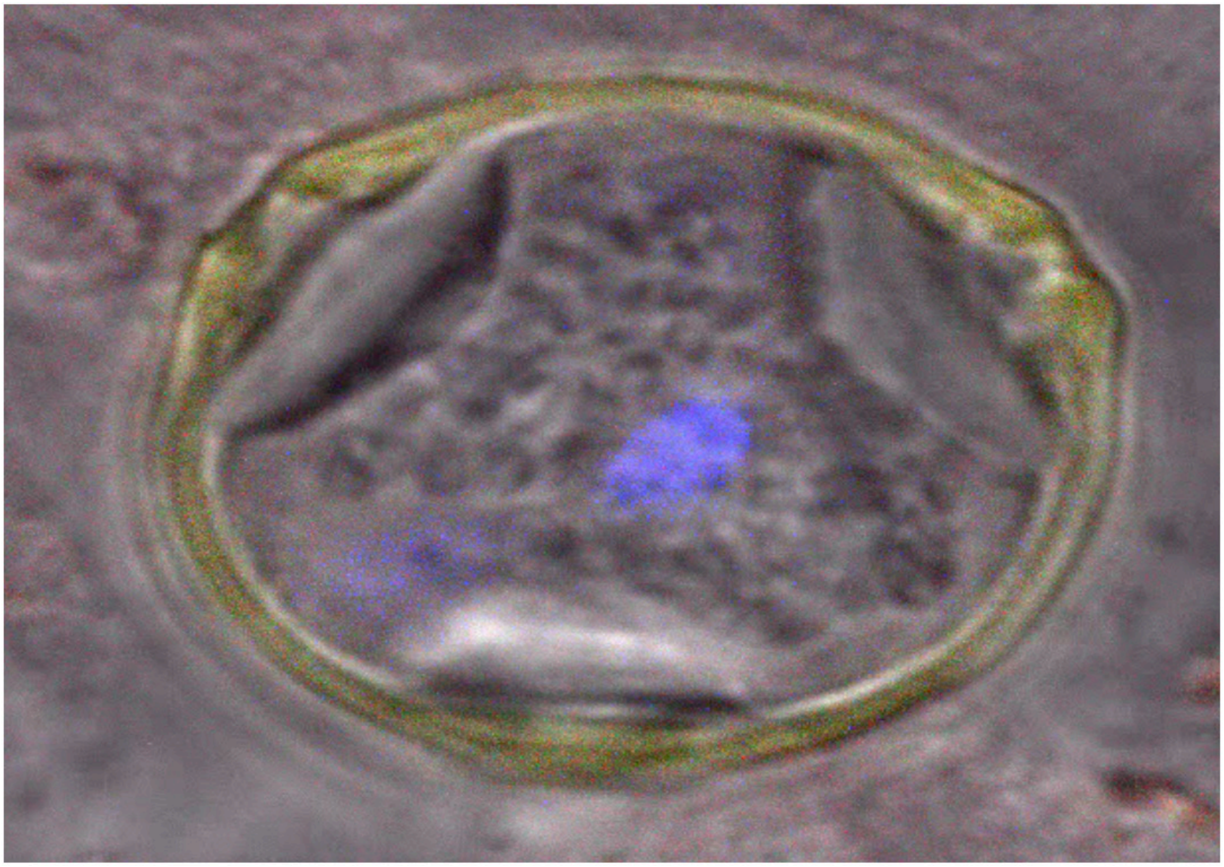

Supplementary Video 2: Scanning of a CICS with 3 pores from the heart of a 45-year-old woman.

See Supplementary File 2

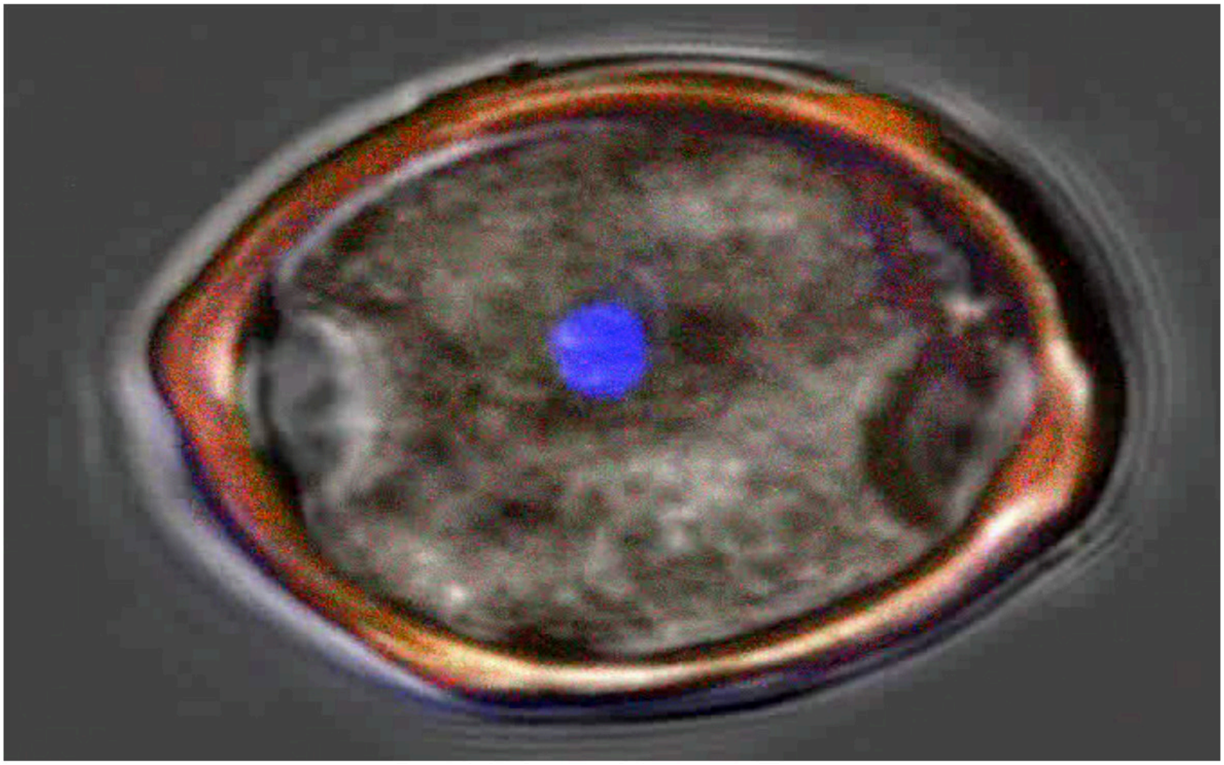

Supplementary Video 3: Scanning of a CICS with 4 pores from the heart of a 45-year-old woman.

See Supplementary File 3

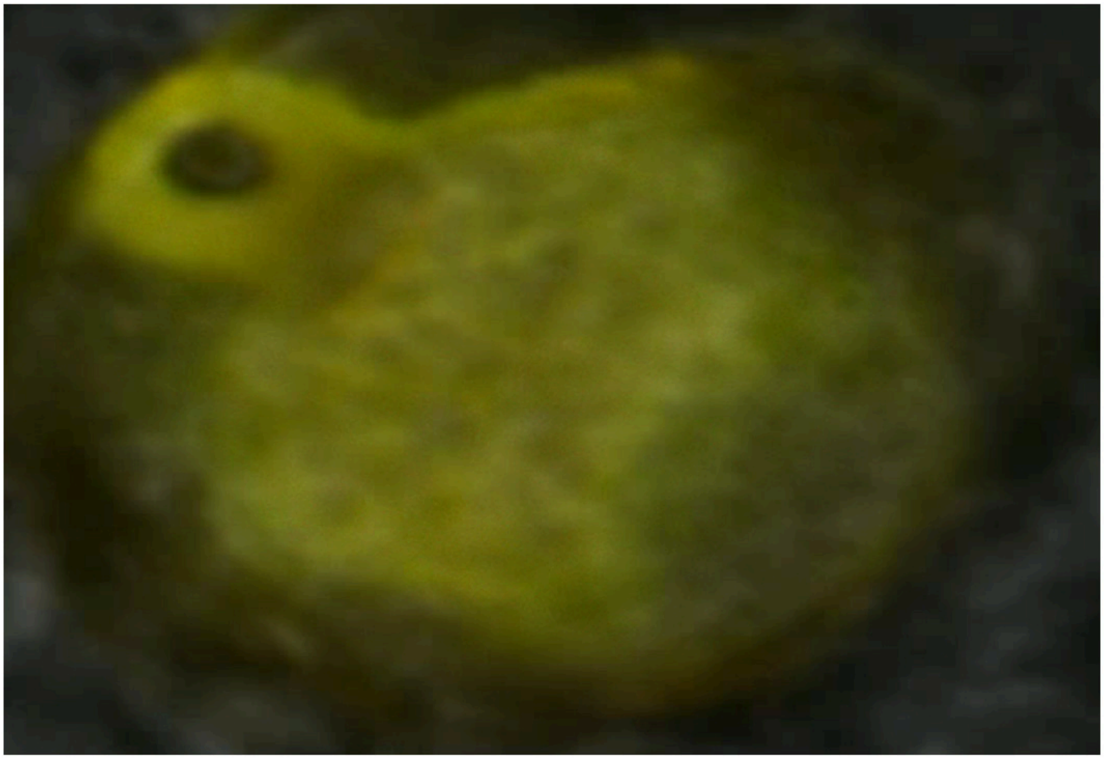

Supplementary Video 4: Scanning of a CICS with 4 pores from the heart of a 40-day-old rat.

See Supplementary File 4

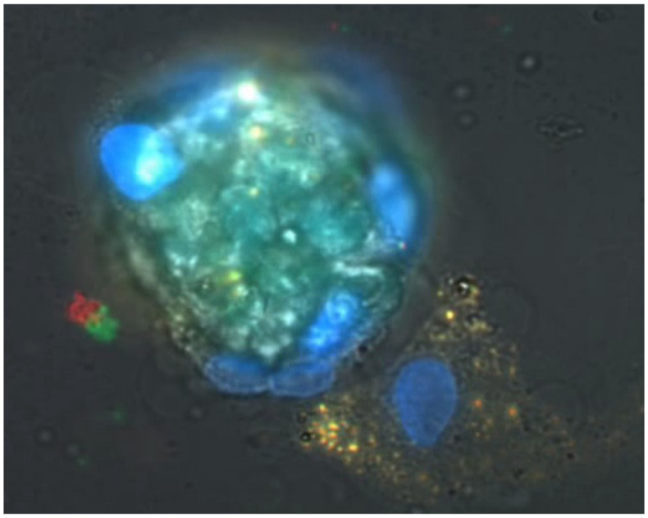

Supplementary Video 5: Scanning of a CICS formed by penetration of a green cell (DiO) into a red cell (Dil). Nuclei are stained by Hoechst.

See Supplementary File 5

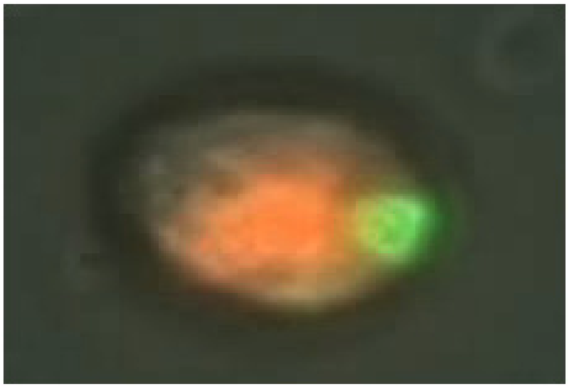

Supplementary Video 6: Proliferation of a small red cell (Dil) inside a large green cell (DiO).

See Supplementary File 6
